# Supplementary figures and images for: Mitochondrial calcium uptake orchestrates vertebrate pigmentation via transcriptional regulation of keratin filaments
Source: PLoS Biol. 2024 Nov 11;22(11):e3002895. doi: 10.1371/journal.pbio.3002895 (PMC11581414; doi:10.1371/journal.pbio.3002895)

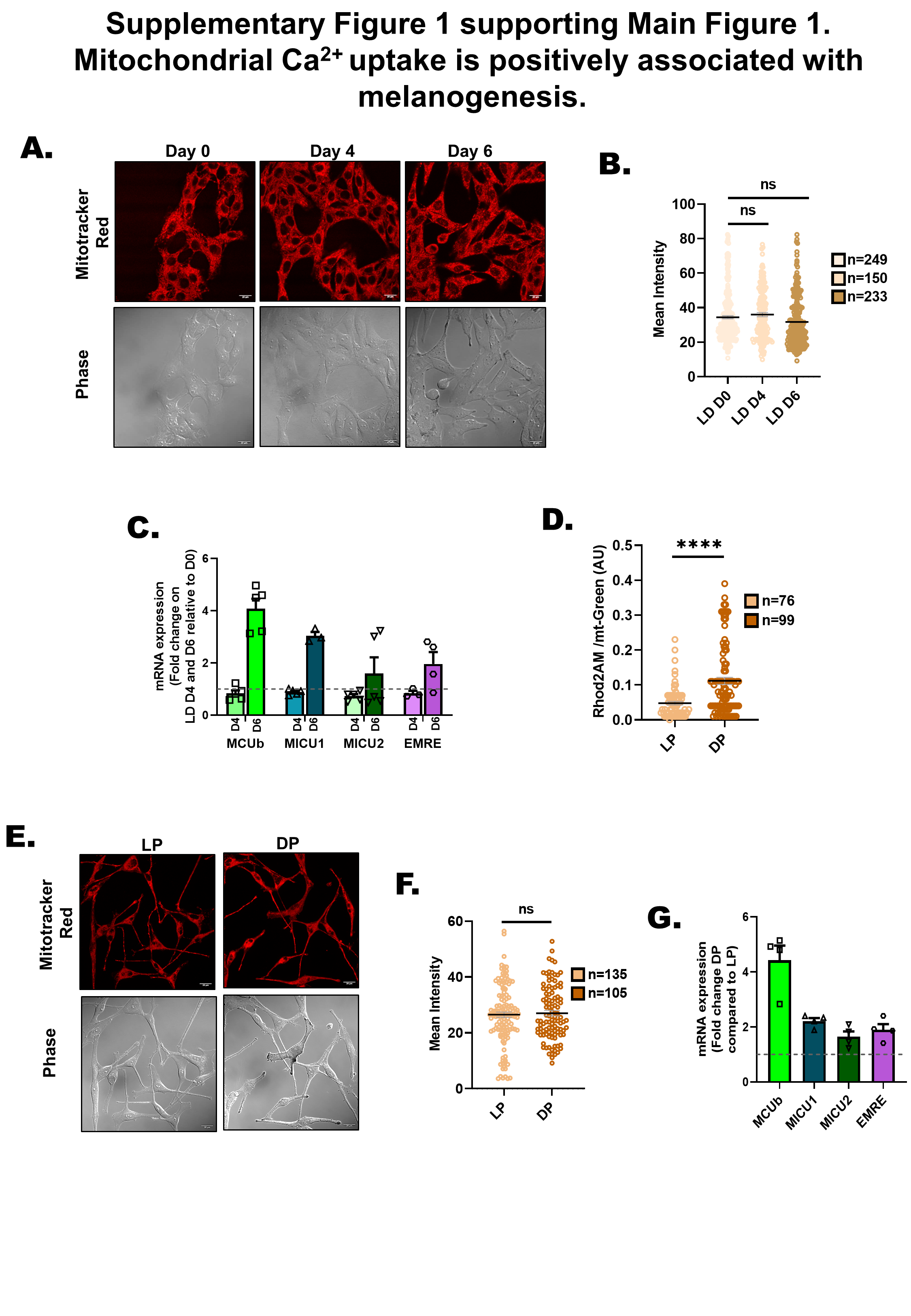

Supplement: S1 Fig — Mitochondrial Ca2+ uptake is positively associated with melanogenesis. (A) Representative confocal microscopy images of Mitotracker-Red staining of mitochondria on LD day 0, LD day 4, and LD day 6 B16 cells. Images have been captured at 63× (oil) magnification in a Zeiss confocal microscope (scale = 20 μm). (B) Quantitation of Mitotracker-Red staining to evaluate mitochondrial content on LD day 0, LD day 4, and LD day 6 B16 cells. (C) qRT-PCR analysis showing relative mRNA expression of MCU complex components (MCUb, MICU1, MICU2, and EMRE) in B16 LD model on LD day 4 and LD day 6 (N = 3–5). (D) Quantitation of Rhod-2/mt-Green in LP and DP primary human melanocytes stimulated with 100 μm histamine where “n” denotes the number of ROIs. (E) Representative confocal microscopy images of Mitotracker-Red staining of mitochondria in LP and DP primary human melanocytes. Images have been captured at 63× (oil) magnification in a Zeiss confocal microscope (scale = 20 μm). (F) Quantitation of Mitotracker-Red staining to evaluate mitochondrial content in LP and DP primary human melanocytes. (G) qRT-PCR analysis showing relative mRNA expression of MCU complex components (MCU, MCUb, MICU1, MICU2, and EMRE) in DP primary human melanocytes in comparison to LP primary human melanocytes (N = 4). Data presented are mean ± SEM. For statistical analysis, unpaired Student’s t test was performed for panels D and F while one-way ANOVA followed by Tukey’s post hoc test was performed for panel B using GraphPad Prism software. Here, ns means nonsignificant; * p < 0.05; ** p < 0.01; *** p < 0.001, and **** p < 0.0001. The data underlying for panels B, C, D, F, and G shown in the figure can be found in S2 Data. (TIF) [file pbio.3002895.s001.TIF]

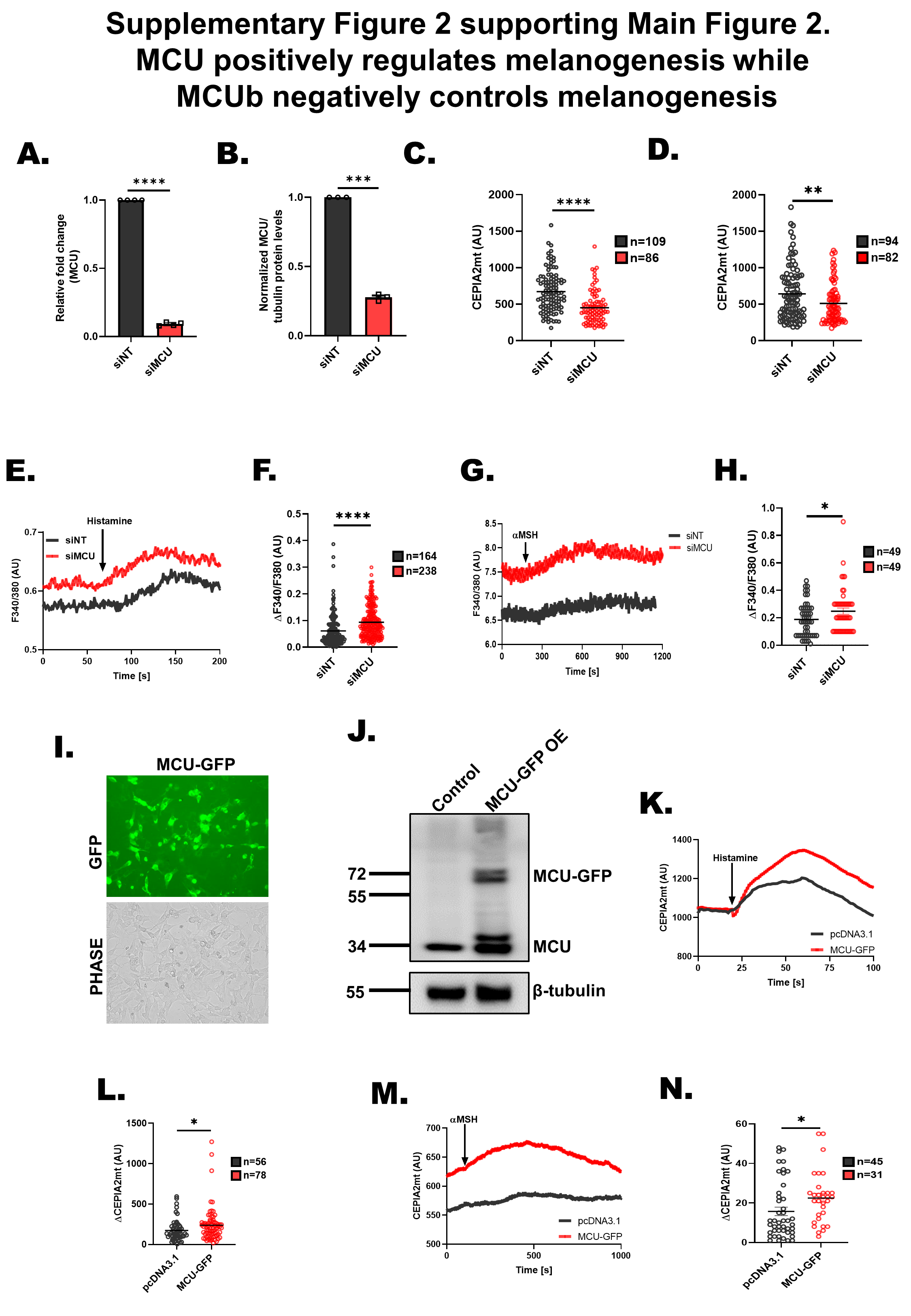

Supplement: S2 Fig — MCU positively regulates melanogenesis while MCUb negatively controls melanogenesis. (A) qRT-PCR analysis showing decrease in MCU mRNA expression upon MCU silencing in B16 cells (N = 4). (B) Densitometric quantitation showing MCU levels on LD day 6 in siNT control and siMCU condition (N = 3). (C) Quantitation of resting mitochondrial Ca2+ with CEPIA2mt in siNT control and siMCU B16 cells stimulated with 100 μm histamine where “n” denotes the number of ROIs. (D) Quantitation of resting mitochondrial Ca2+ with CEPIA2mt in siNT control and siMCU B16 cells stimulated with 1 μm αMSH where “n” denotes the number of ROIs. (E) Representative traces of Fura-2 imaging to measure cytosolic Ca2+ in siNon-Targeting (siNT) control and siMCU B16 cells stimulated with 100 μm histamine. (F) Quantitation of cytosolic Ca2+ levels in siNT control and siMCU B16 cells stimulated with 100 μm histamine where “n” denotes the number of ROIs (cytosolic Ca2+ levels in siNT control, siMCU and siMCUb were measured on same day). (G) Representative traces of Fura-2 imaging to measure cytosolic Ca2+ in siNon-Targeting (siNT) control and siMCU B16 cells stimulated with 1 μm αMSH. (H) Quantitation of cytosolic Ca2+ levels in siNT control and siMCU B16 cells stimulated with 1 μm αMSH where “n” denotes the number of ROIs. (I) Representative GFP and bright field images showing MCU-GFP transfected B16 cells (scale = 100 μm) (N = 3). (J) Representative western blot demonstrating ectopic expression of GFP-tagged human MCU in B16 cells (N = 3). (K) Representative mitochondrial Ca2+ imaging traces of pcDNA control plasmid and MCU-GFP overexpressing B16 cells stimulated with 100 μm histamine. (L) Quantitation of mitochondrial Ca2+ uptake by calculating increase in CEPIA2mt signal (ΔCEPIA2mt) in pcDNA control plasmid and MCU-GFP overexpressing B16 cells upon stimulation with 100 μm histamine where “n” denotes the number of ROIs. (M) Representative mitochondrial Ca2+ imaging traces of pcDNA control plasmid and [file pbio.3002895.s002.TIF]

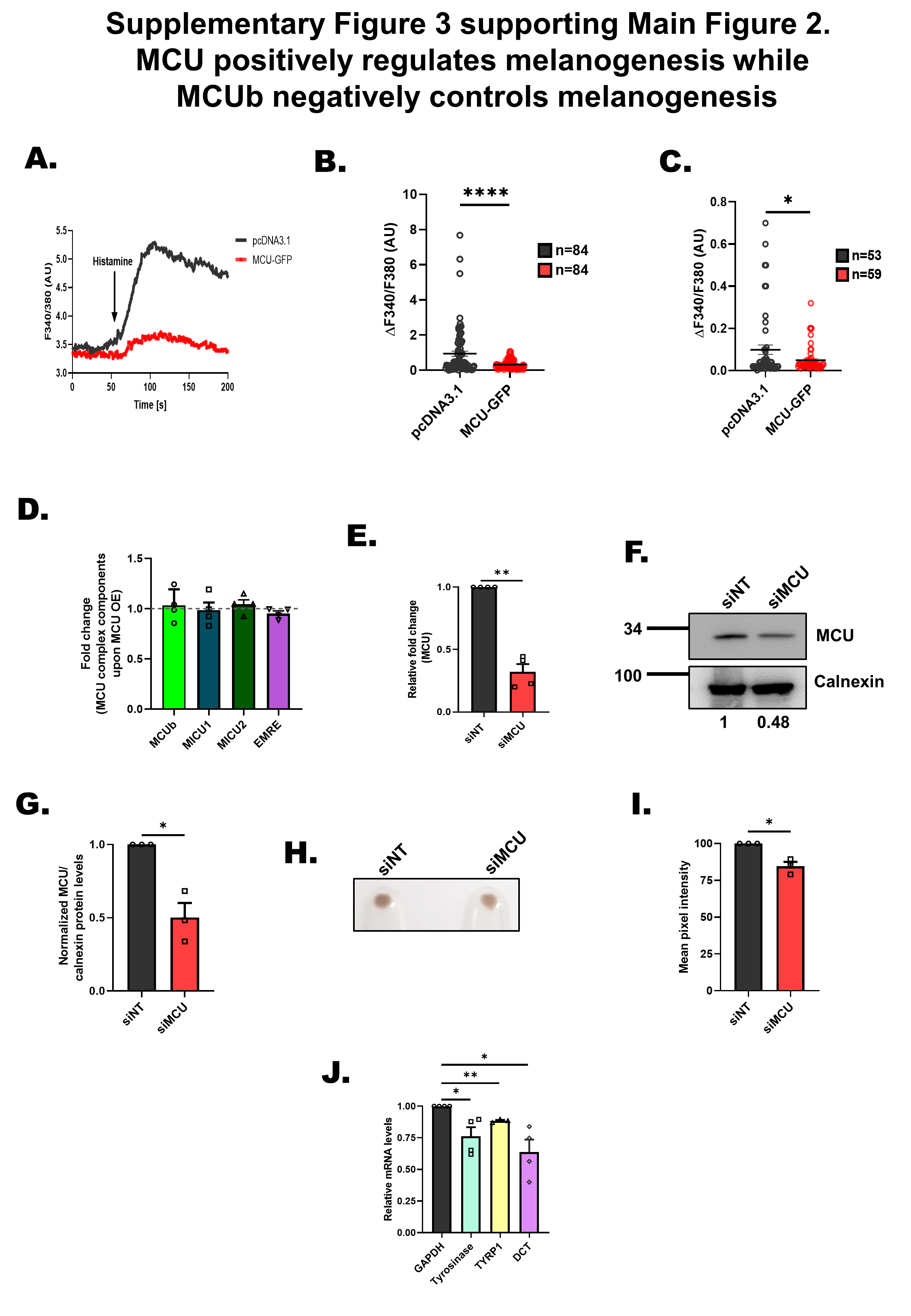

Supplement: S3 Fig — MCU positively regulates melanogenesis while MCUb negatively controls melanogenesis. (A) Representative traces of Fura-2 imaging to measure cytosolic Ca2+ in pcDNA control plasmid and MCU-GFP overexpressing B16 cells stimulated with 100 μm histamine. (B) Quantitation of cytosolic Ca2+ levels in pcDNA control plasmid and MCU-GFP overexpressing B16 cells stimulated with 100 μm histamine where “n” denotes the number of ROIs. (C) Quantitation of cytosolic Ca2+ levels in pcDNA control plasmid and MCU-GFP overexpressing B16 cells stimulated with 1 μm αMSH where “n” denotes the number of ROIs. (D) qRT–PCR analysis showing relative mRNA expression of MCU complex components (MCUb, MICU1, MICU2, and EMRE) upon MCU-GFP overexpression. (E) qRT-PCR analysis showing decrease in MCU mRNA expression upon MCU silencing in primary human melanocytes (N = 4). (F) Representative western blot confirming siRNA based silencing of MCU in primary human melanocytes. Densitometric analysis using ImageJ is presented below the blot (N = 3). (G) Densitometric quantitation showing MCU levels in siNT control and siMCU condition in primary human melanocytes (N = 3). (H) Representative pellet images of siNT control and siMCU in primary human melanocytes (N = 4). (I) Mean pixel intensity of siNT control and siMCU pellet images in primary human melanocytes (N = 3). (J) qRT-PCR analysis showing decrease in Tyrosinase, TYRP1, and DCT mRNA expression upon MCU silencing in primary human melanocytes (N = 4). Data presented are mean ± SEM. For statistical analysis, unpaired Student’s t test was performed for panels B and C while one sample t test was performed for panels E, G, I, and J using GraphPad Prism software. * p < 0.05; ** p < 0.01; and **** p < 0.0001. The data underlying for panels A–E, G, I, and J shown in the figure can be found in S2 Data. (TIF) [file pbio.3002895.s003.TIF]

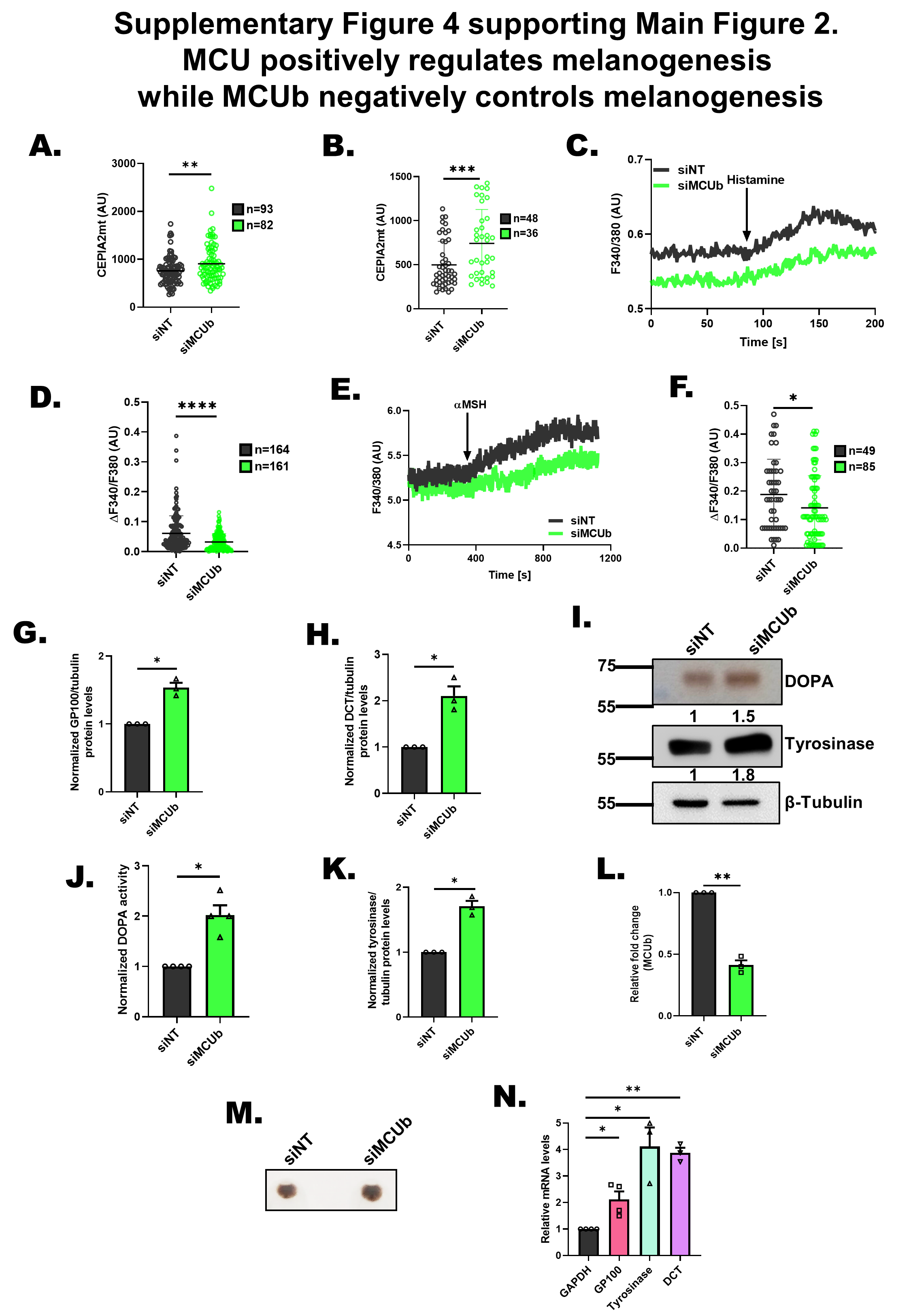

Supplement: S4 Fig — MCU positively regulates melanogenesis while MCUb negatively controls melanogenesis. (A) Quantitation of resting mitochondrial Ca2+ with CEPIA2mt in siNT control and siMCUb B16 cells stimulated with 100 μm histamine. Here, “n” denotes the number of ROIs. (B) Quantitation of resting mitochondrial Ca2+ with CEPIA2mt in siNT control and siMCUb B16 cells stimulated with 1 μm αMSH. Here, “n” denotes the number of ROIs. (C) Representative traces of Fura-2 imaging to measure cytosolic Ca2+ in siNon-Targeting (siNT) control and siMCUb B16 cells stimulated with 100 μm histamine. (D) Quantitation of cytosolic Ca2+ levels in siNT control and siMCUb B16 cells stimulated with 100 μm histamine where “n” denotes the number of ROIs (cytosolic Ca2+ levels in siNT control, siMCU and siMCUb were measured on same day). (E) Representative traces of Fura-2 imaging to measure cytosolic Ca2+ in siNon-Targeting (siNT) control and siMCUb B16 cells stimulated with 1 μm αMSH. (F) Quantitation of cytosolic Ca2+ levels in siNT control and siMCUb B16 cells stimulated with 1 μm αMSH where “n” denotes the number of ROIs. (G) Densitometric quantitation showing GP100 levels on LD day 6 in siNT control and siMCUb condition (N = 3). (H) Densitometric quantitation showing DCT levels on LD day 6 in siNT control and siMCUb condition (N = 3). (I) DOPA assay showing activity of tyrosinase enzyme (N = 4) and representative western blot for tyrosinase expression (N = 3) on LD day 6 upon MCUb silencing as compared to non-targeting control. Densitometric analysis using ImageJ is presented below the blot. (J) Densitometric quantitation showing activity of tyrosinase enzyme on LD day 6 in siNT control and siMCUb condition (N = 4). (K) Densitometric quantitation showing Tyrosinase levels on LD day 6 in siNT control and siMCUb condition (N = 3). (L) qRT-PCR analysis showing decrease in MCUb mRNA expression upon MCUb silencing in LP primary human melanocytes (N = 3). (M) Representative pellet images of siNT control [file pbio.3002895.s004.TIF]

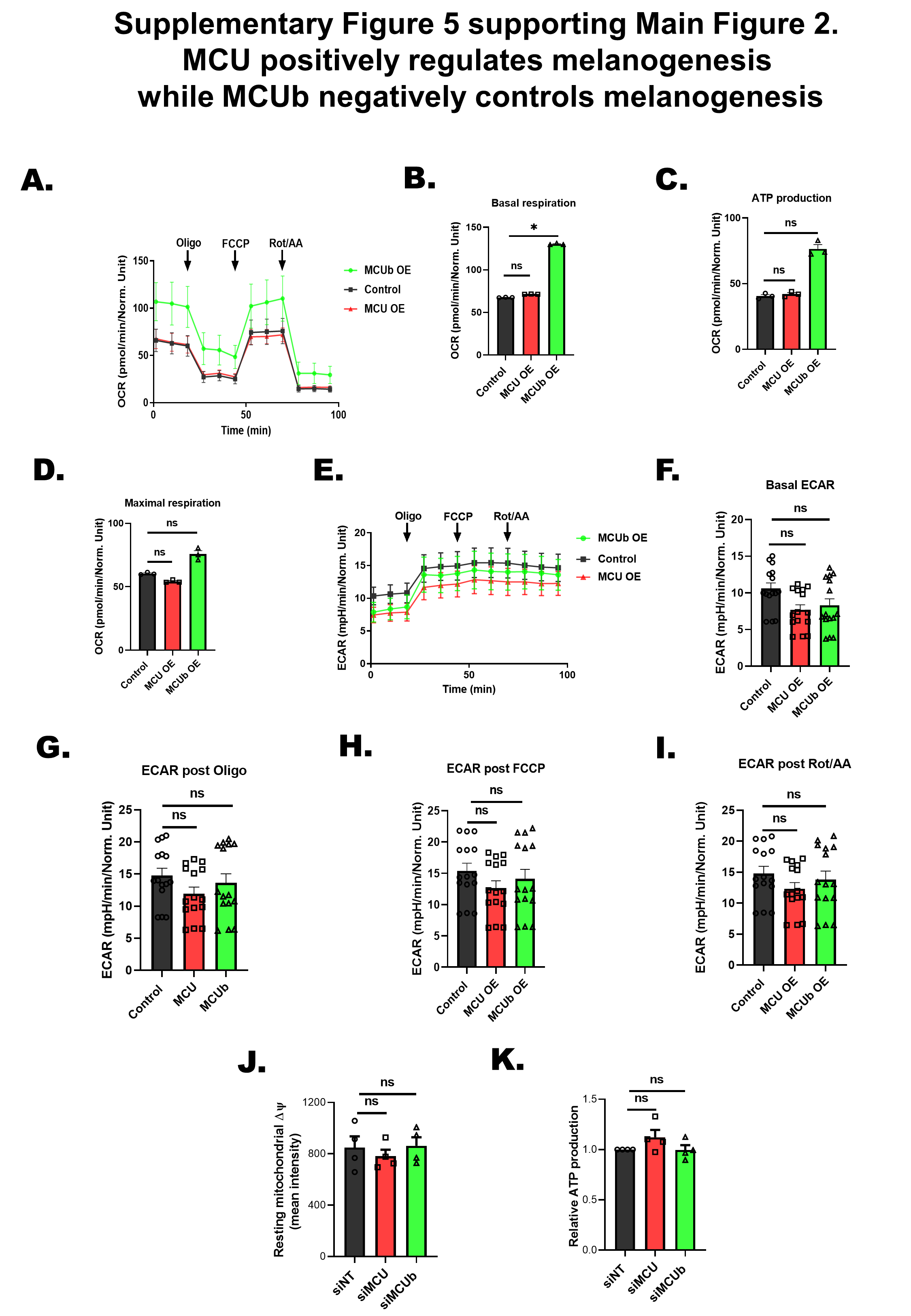

Supplement: S5 Fig — MCU positively regulates melanogenesis while MCUb negatively controls melanogenesis. (A) Oxygen consumption rate (OCR) in control, MCU and MCUb overexpressing B16 cells (2 independent biologicals, with 3 technical replicates in each set). (B) Quantitative analysis of basal respiration in control, MCU and MCUb overexpressing B16 cells. (C) Quantitative analysis of ATP production in control, MCU and MCUb overexpressing B16 cells. (D) Quantitative analysis of maximal respiration in control, MCU and MCUb overexpressing B16 cells. (E) Extracellular acidification rate (ECAR) in control, MCU and MCUb overexpressing B16 cells. (F) Quantitative analysis of basal ECAR in control, MCU and MCUb overexpressing B16 cells. (G) Quantitative analysis of ECAR in control, MCU and MCUb overexpressing B16 cells post oligomycin. (H) Quantitative analysis of ECAR in control, MCU and MCUb overexpressing B16 cells post FCCP. (I) Quantitative analysis of ECAR in control, MCU and MCUb overexpressing B16 cells post rotenone/antimycin A. (J) Resting mitochondrial membrane potential (ΔΨ), measured with TMRE in siNT control, siMCU and siMCUb B16 cells (N = 5). (K) Mitochondrial ATP levels in siNT control, siMCU and siMCUb B16 cells (N = 4). Data presented are mean ± SEM. For statistical analysis, Kruskal–Wallis test was performed for panels B, C, D, F, G, I, and H, while one-way ANOVA followed by Dunnette’s multiple comparisons test was performed for panel J and K using GraphPad Prism software. Here, ns means nonsignificant. The data underlying for panels A–K shown in the figure can be found in S2 Data. (TIF) [file pbio.3002895.s005.TIF]

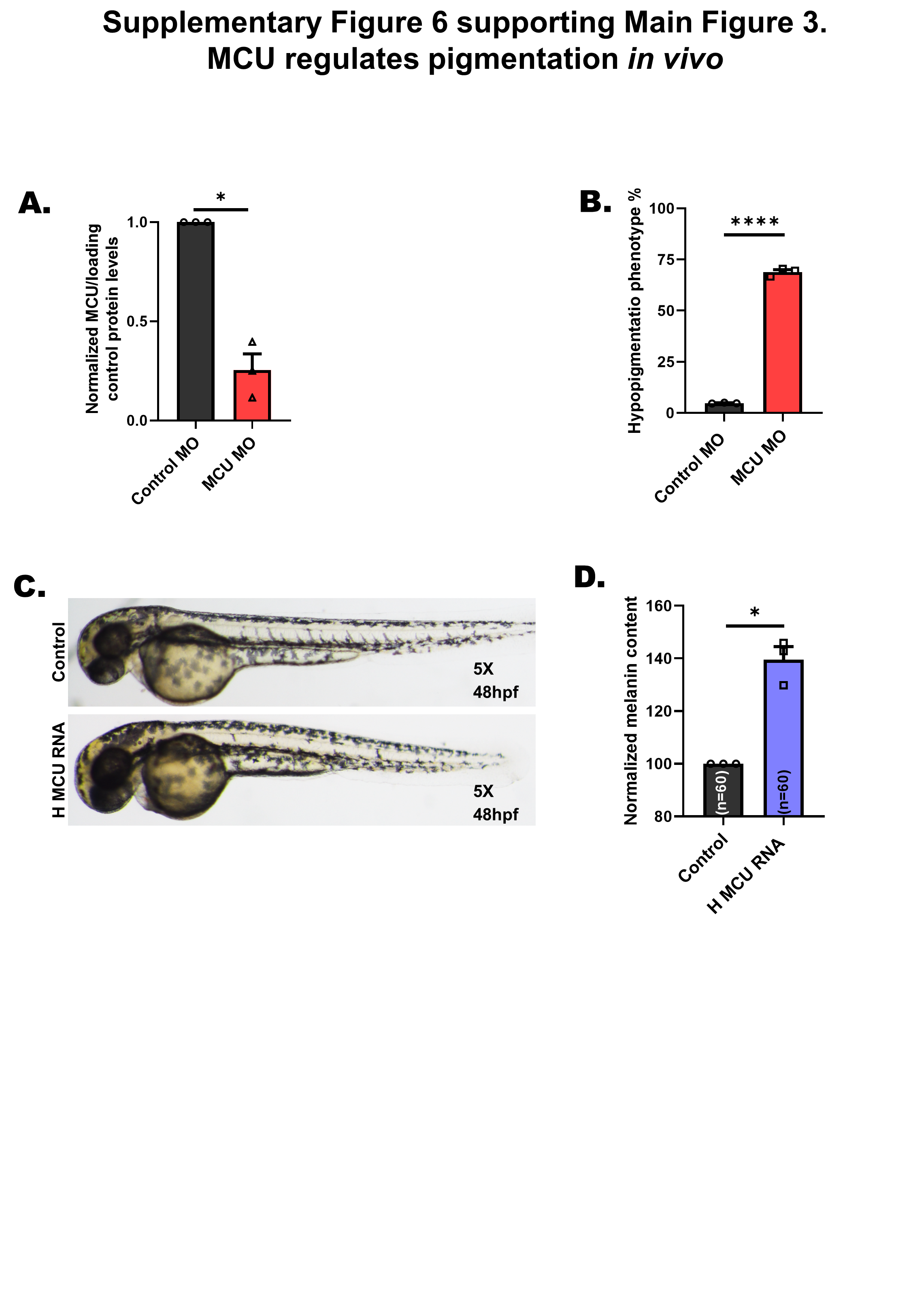

Supplement: S6 Fig — MCU regulates pigmentation in vivo. (A) Densitometric quantitation showing MCU levels in control MO and MCU MO (N = 3). (B) The hypopigmentation phenotype analyzed at 30 hpf in around 200 zebrafish embryos from 3 independent sets of injections (N = 3 independent experiments with approximately 200 embryos/condition). (C) Representative bright-field images of zebrafish embryos injected with either control or human MCU RNA at 48 hpf (N = 3 independent experiments with approximately 200 embryos/condition). (D) Melanin-content estimation of zebrafish embryos injected with either control or human MCU RNA injection in 60 zebrafish embryos from 3 independent sets of injections (N = 3 independent experiments with 60 embryos/condition). Data presented are mean ± SEM. For statistical analysis, unpaired Student’s t test was performed for panel B, while one sample t test was performed for panels A and D using GraphPad Prism software. Here, * p < 0.05; **** p < 0.0001. The data underlying for panels A, B, and D shown in the figure can be found in S2 Data. (TIF) [file pbio.3002895.s006.TIF]

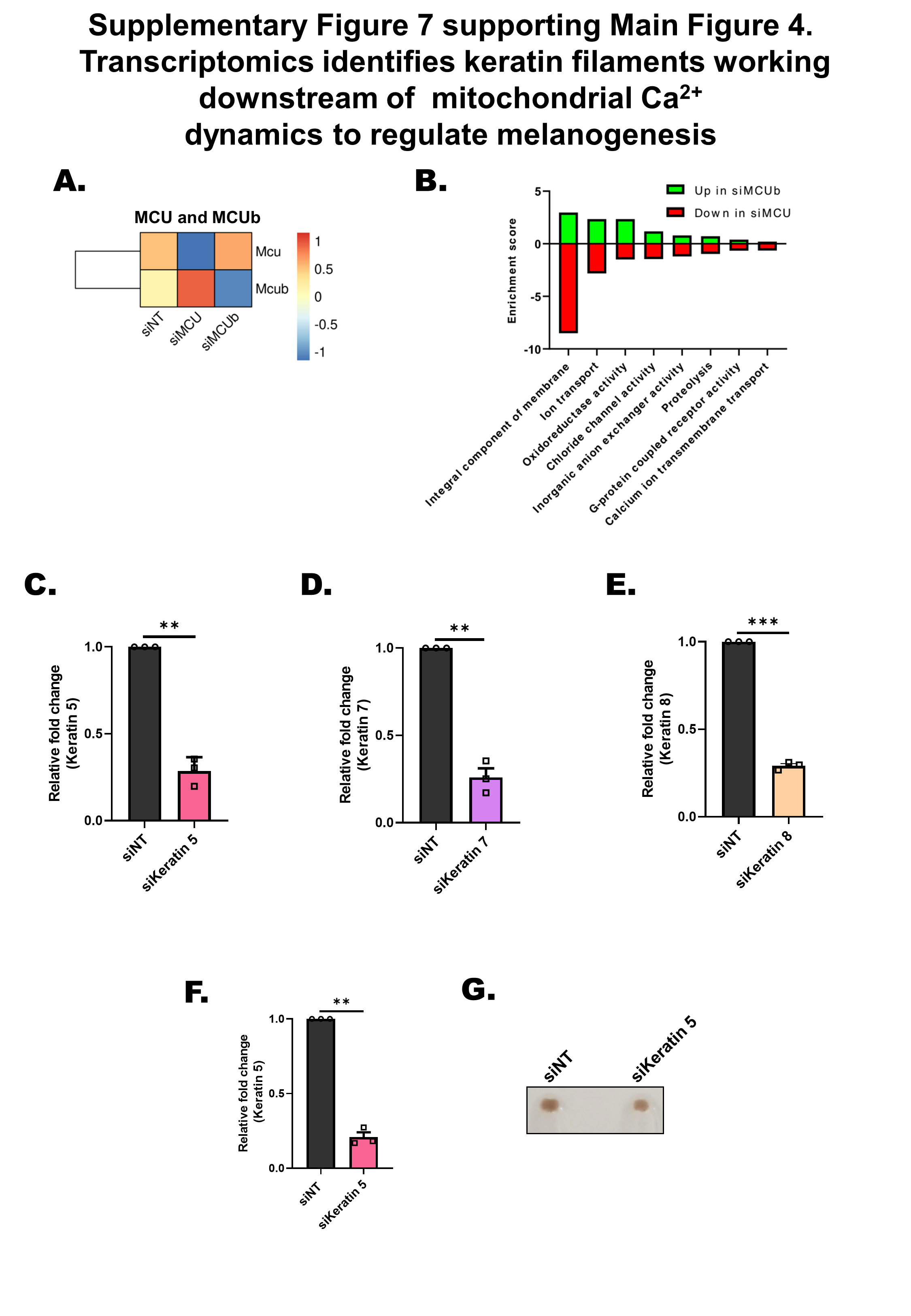

Supplement: S7 Fig — Transcriptomics identifies keratin filaments working downstream of mitochondrial Ca2+ dynamics to regulate melanogenesis. (A) Heatmap representing the expression of MCU and MCUb upon silencing of MCU and MCUb, respectively. Scale from blue to red represents z-score for fold change from −1 to +1. (B) Common oppositely regulated pathways down in siMCU and up in siMCUb. (C) qRT-PCR analysis showing decrease in keratin 5 mRNA expression upon keratin 5 silencing in B16 cells (N = 3). (D) qRT-PCR analysis showing decrease in keratin 7 mRNA expression upon keratin 7 silencing in B16 cells (N = 3). (E) qRT-PCR analysis showing decrease in keratin 8 mRNA expression upon keratin 8 silencing in B16 cells (N = 3). (F) qRT-PCR analysis showing decrease in Keratin 5 mRNA expression upon Keratin 5 silencing in LP primary human melanocytes (N = 3). (G) Representative pellet images of siNT control and siKeratin 5 in LP primary human melanocytes (N = 3). Data presented are mean ± SEM. For statistical analysis, one sample t test was performed for panels C–F using GraphPad Prism software. Here, ** p < 0.01 and *** p < 0.001. The data underlying for panels A–F shown in the figure can be found in S2 Data. (TIF) [file pbio.3002895.s007.TIF]

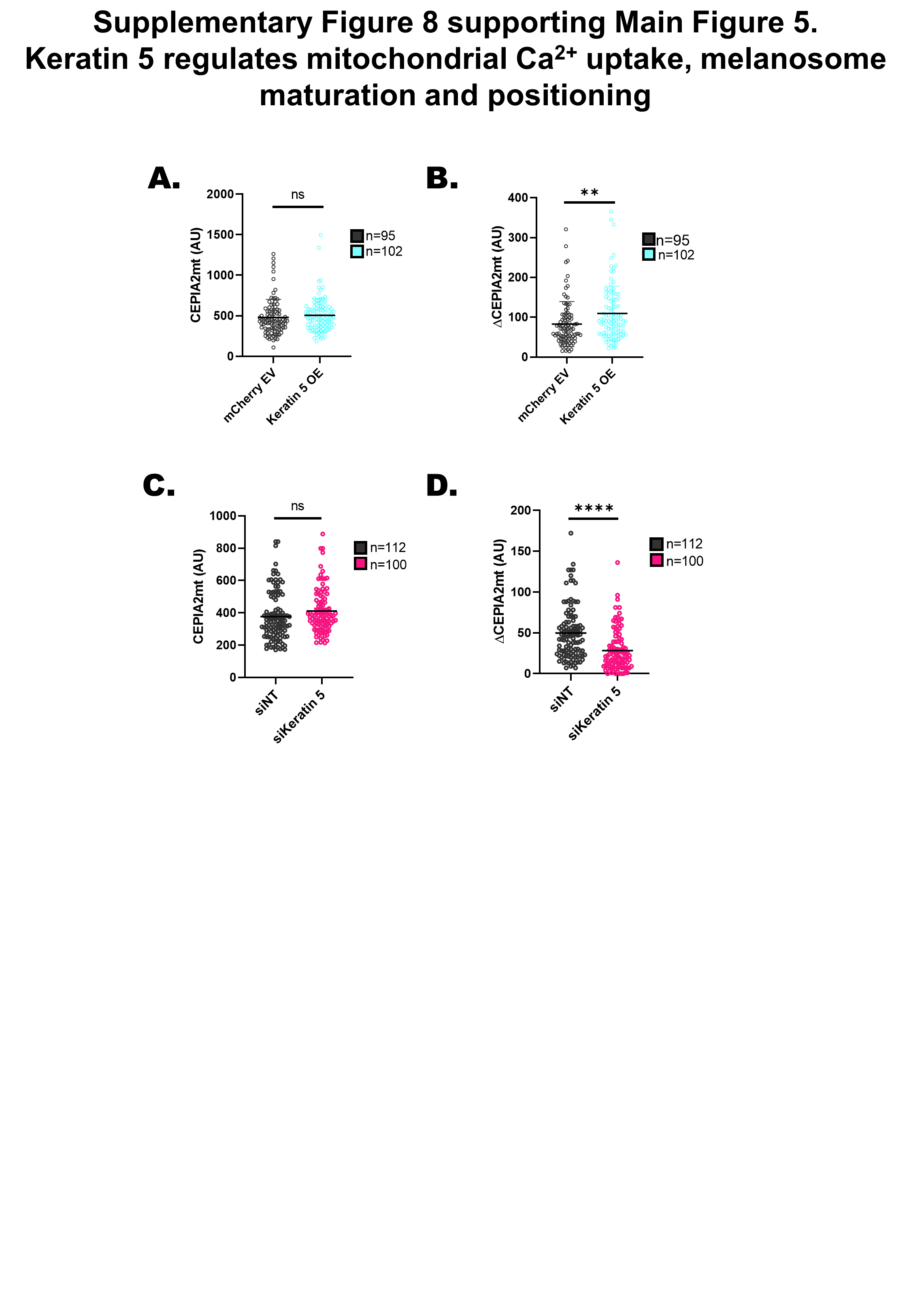

Supplement: S8 Fig — Keratin 5 regulates mitochondrial Ca2+ uptake, melanosome maturation, and positioning. (A) Quantitation of resting mitochondrial Ca2+ with CEPIA2mt in mCherry empty vector (EV) control and Keratin 5 overexpressing (OE) cells where “n” denotes the number of ROIs. (B) Quantitation of mitochondrial Ca2+ uptake by calculating increase in CEPIA2mt signal (ΔCEPIA2mt) in mCherry empty vector (EV) control and Keratin 5 overexpressing (OE) cells upon stimulation with 100 μm histamine where “n” denotes the number of ROIs. (C) Quantitation of resting mitochondrial Ca2+ with CEPIA2mt in siNT control and siKeratin 5 cells where “n” denotes the number of ROIs. (D) Quantitation of mitochondrial Ca2+ uptake by calculating increase in CEPIA2mt signal (ΔCEPIA2mt) in siNT control and siKeratin 5 cells upon stimulation with 100 μm histamine where “n” denotes the number of ROIs. Data presented are mean ± SEM. For statistical analysis, unpaired Student’s t test was performed for panels A–D using GraphPad Prism software. Here, ** p < 0.01; **** p < 0.0001. The data underlying for panels A–D shown in the figure can be found in S2 Data. (TIF) [file pbio.3002895.s008.TIF]

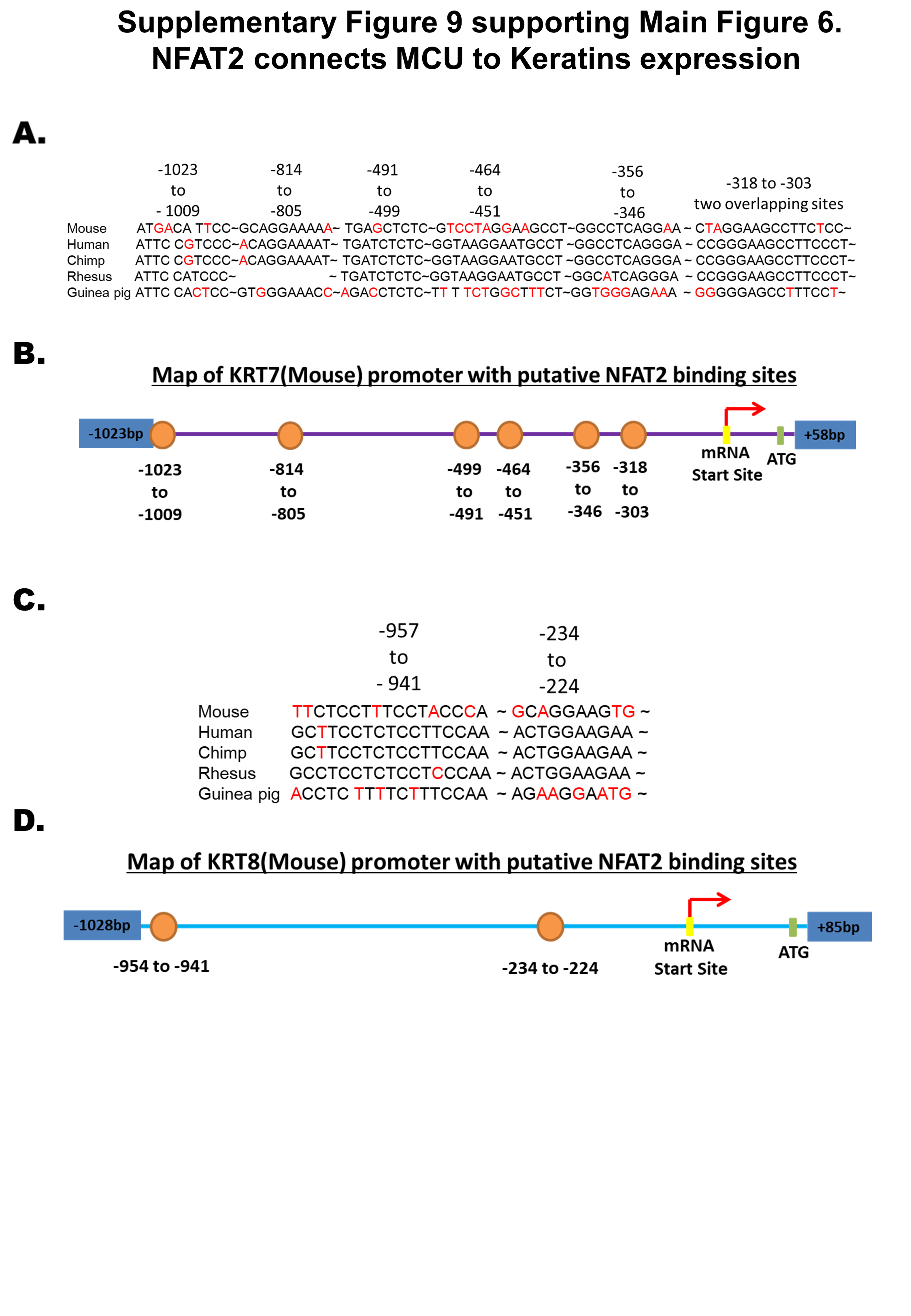

Supplement: S9 Fig — NFAT2 connects MCU to keratins expression. (A) Multispecies sequence alignment of putative NFAT2 binding sites in the mouse keratin 7 (KRT7) core promoter. (B) Schematic representation of putative NFAT2 binding sites in the mouse KRT7 core promoter. (C) Multispecies sequence alignment of putative NFAT2 binding sites in the mouse keratin 8 (KRT8) core promoter. (D) Schematic representation of putative NFAT2 binding sites in the mouse KRT8 core promoter. (TIF) [file pbio.3002895.s009.TIF]

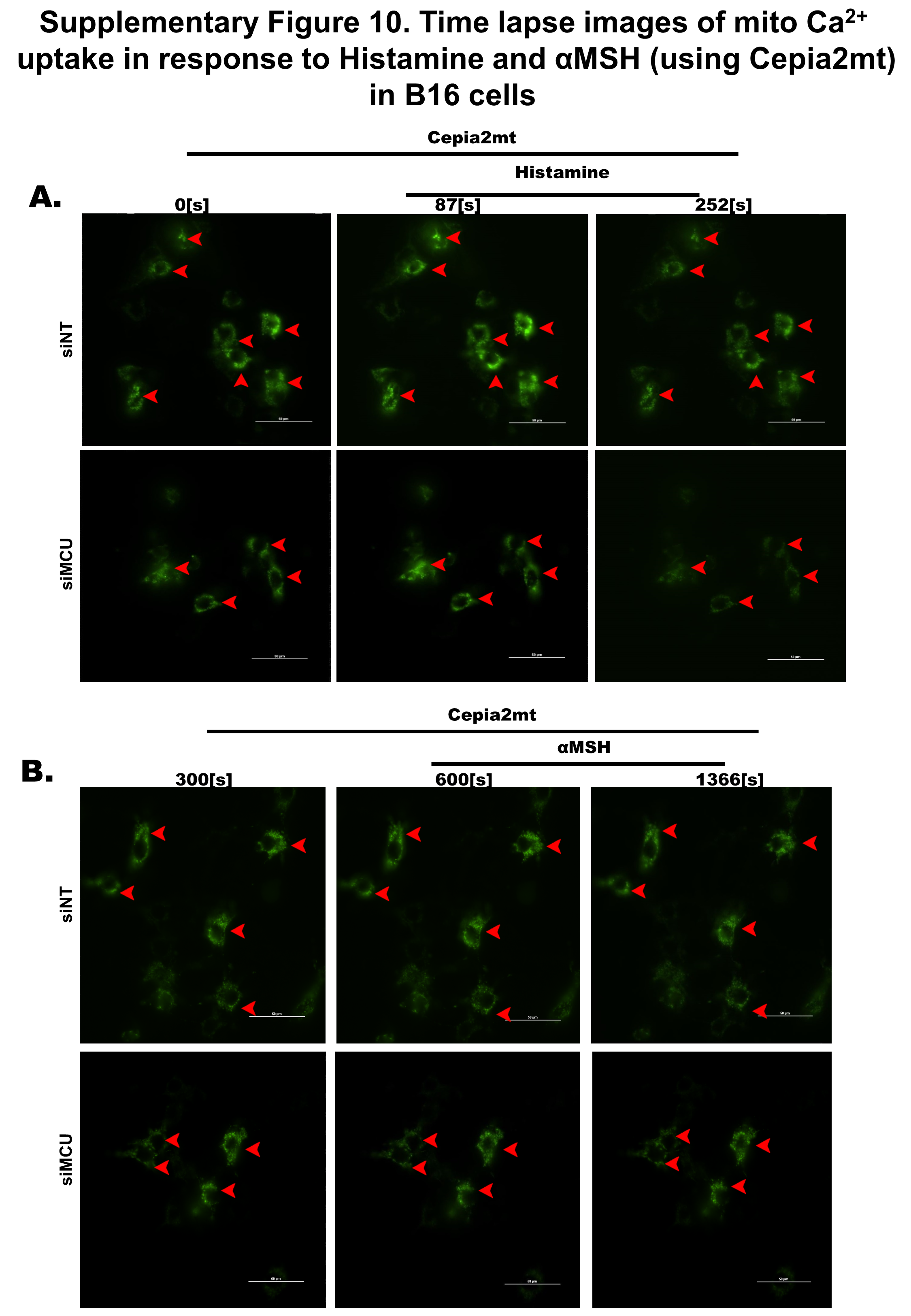

Supplement: S10 Fig — (A) Time lapse images of mitochondrial Ca2+ uptake with CEPIA2mt in siNT control and siMCU B16 cells stimulated with 100 μm histamine (scale = 50 μm). (B) Time lapse images of mitochondrial Ca2+ uptake with CEPIA2mt in siNT control and siMCU B16 cells stimulated with 1 μm αMSH (scale = 50 μm). Arrows indicate change in Cepia2mt signal in cells. (TIF) [file pbio.3002895.s010.TIF]

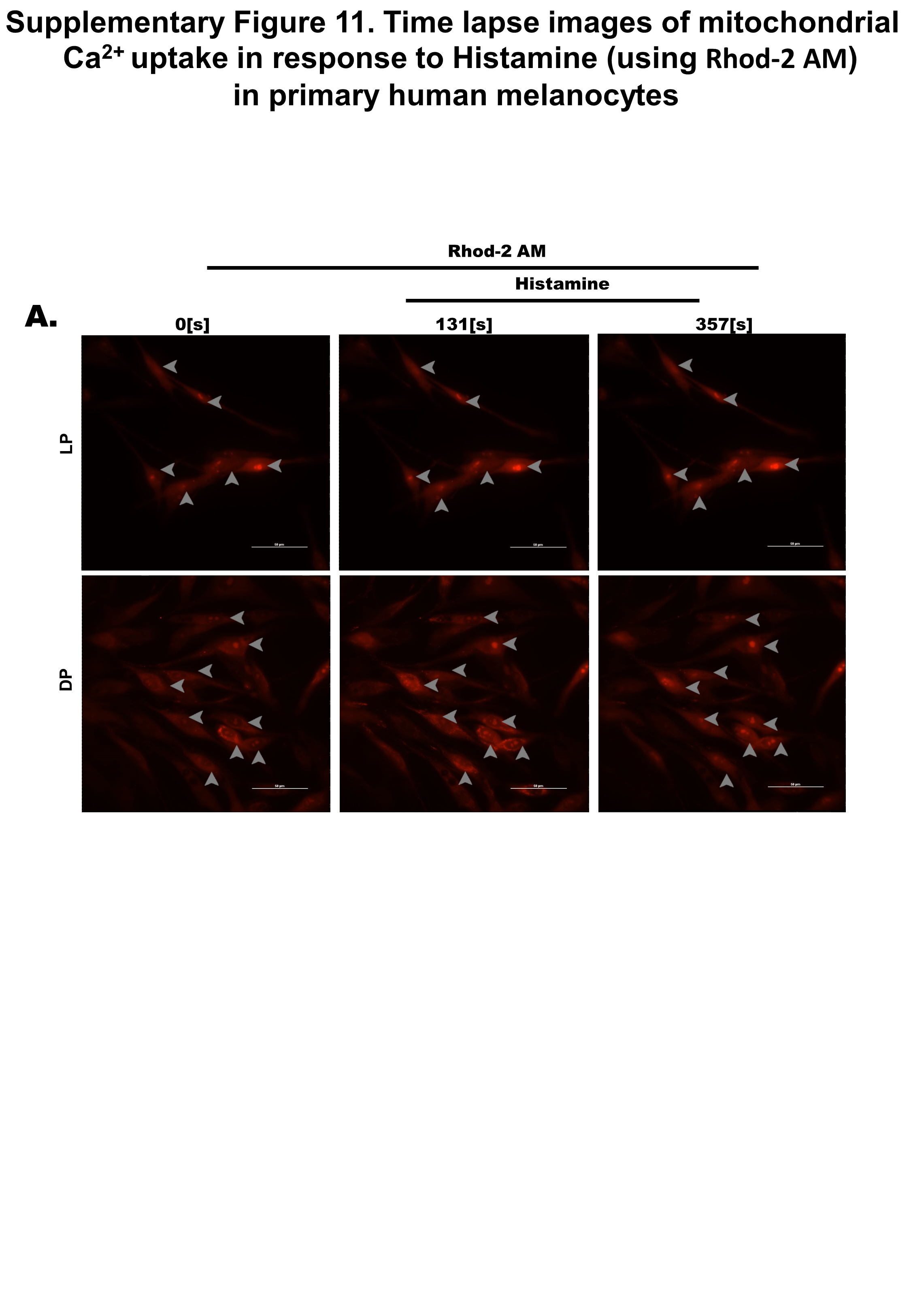

Supplement: S11 Fig — (A) Time lapse images of mitochondrial Ca2+ uptake with Rhod-2 AM in LP and DP primary melanocytes stimulated with 100 μm histamine (scale = 50 μm). Arrows indicate change in Rhod 2AM signal in cells. (TIF) [file pbio.3002895.s011.TIF]

**Fig 1G**

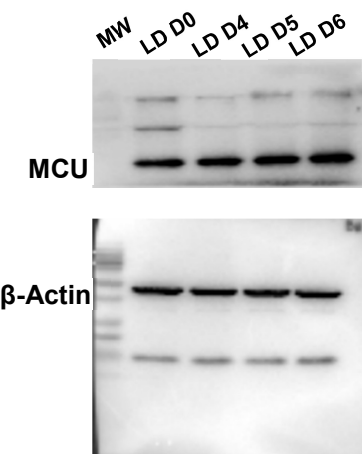

**Fig 1O**

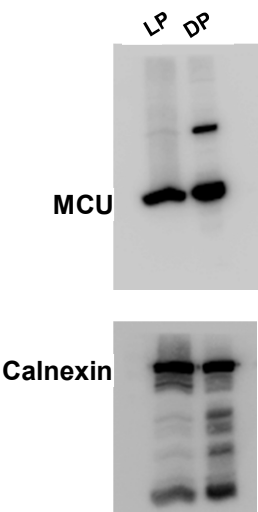

**Fig 2B**

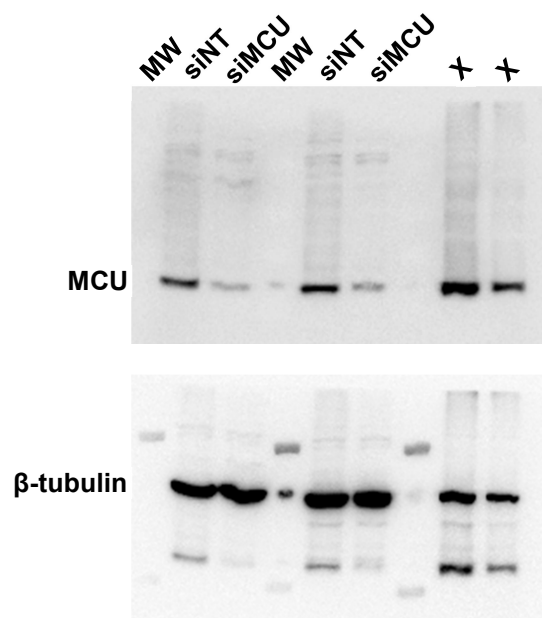

**Fig 2S**

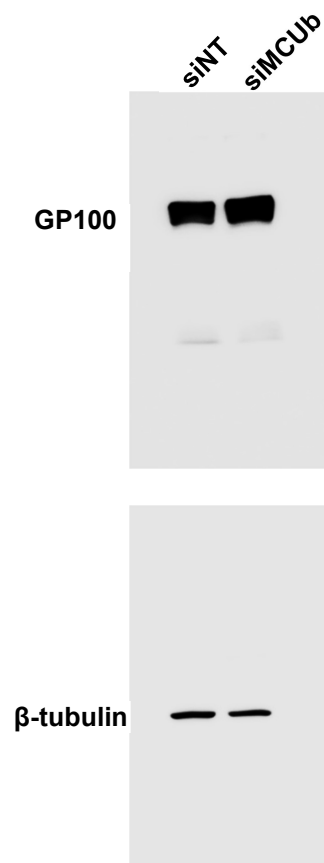

**Fig 2S**

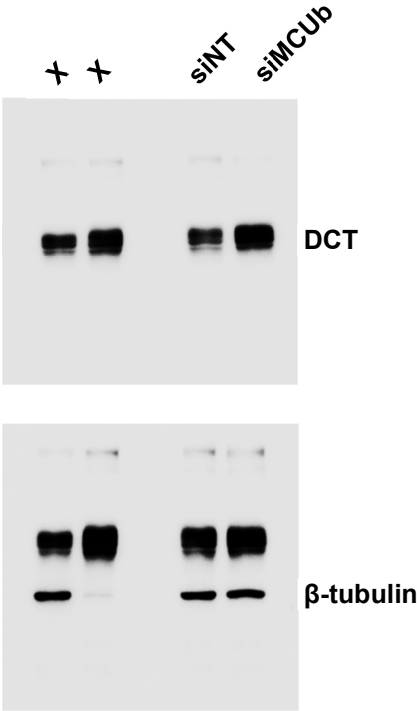

**Fig 3A**

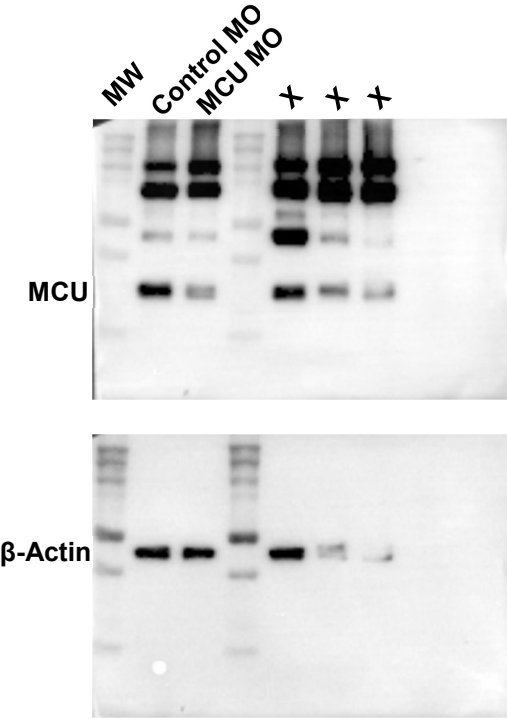

**Fig 5I**

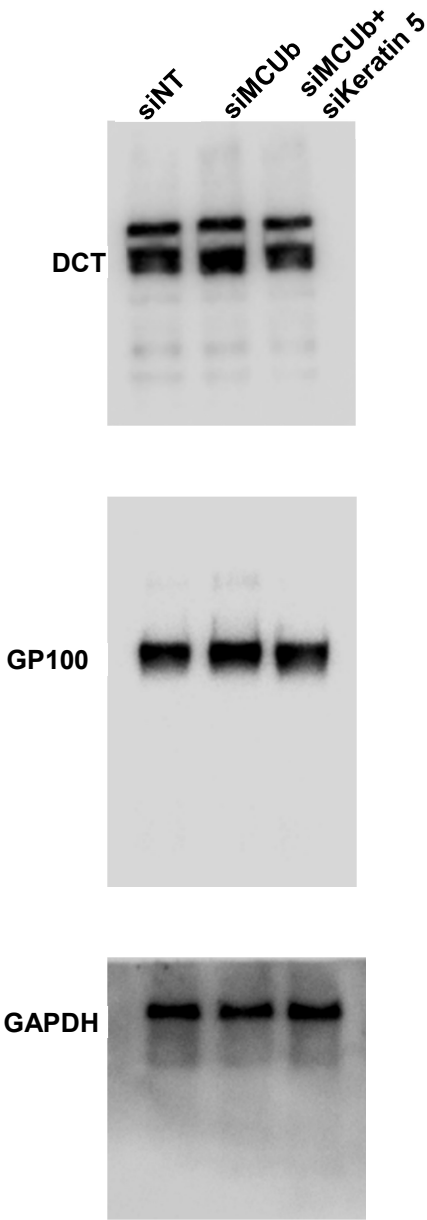

**S2J Fig**

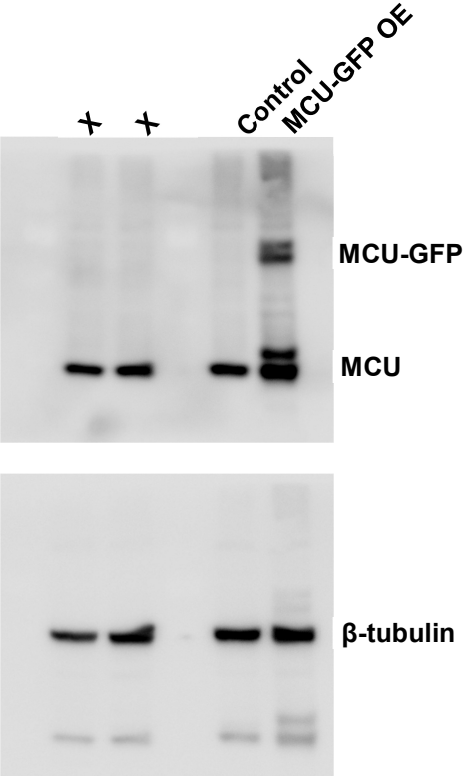

**S3F Fig**

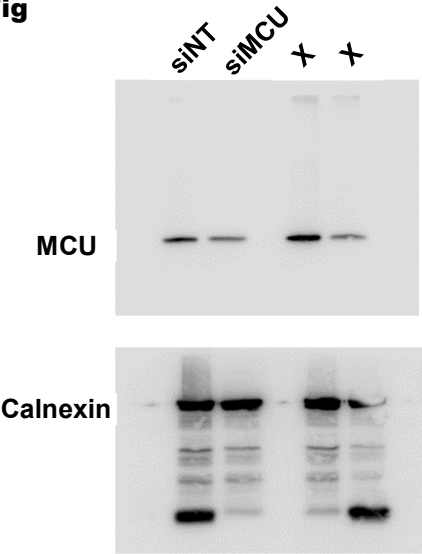

**S4I Fig**

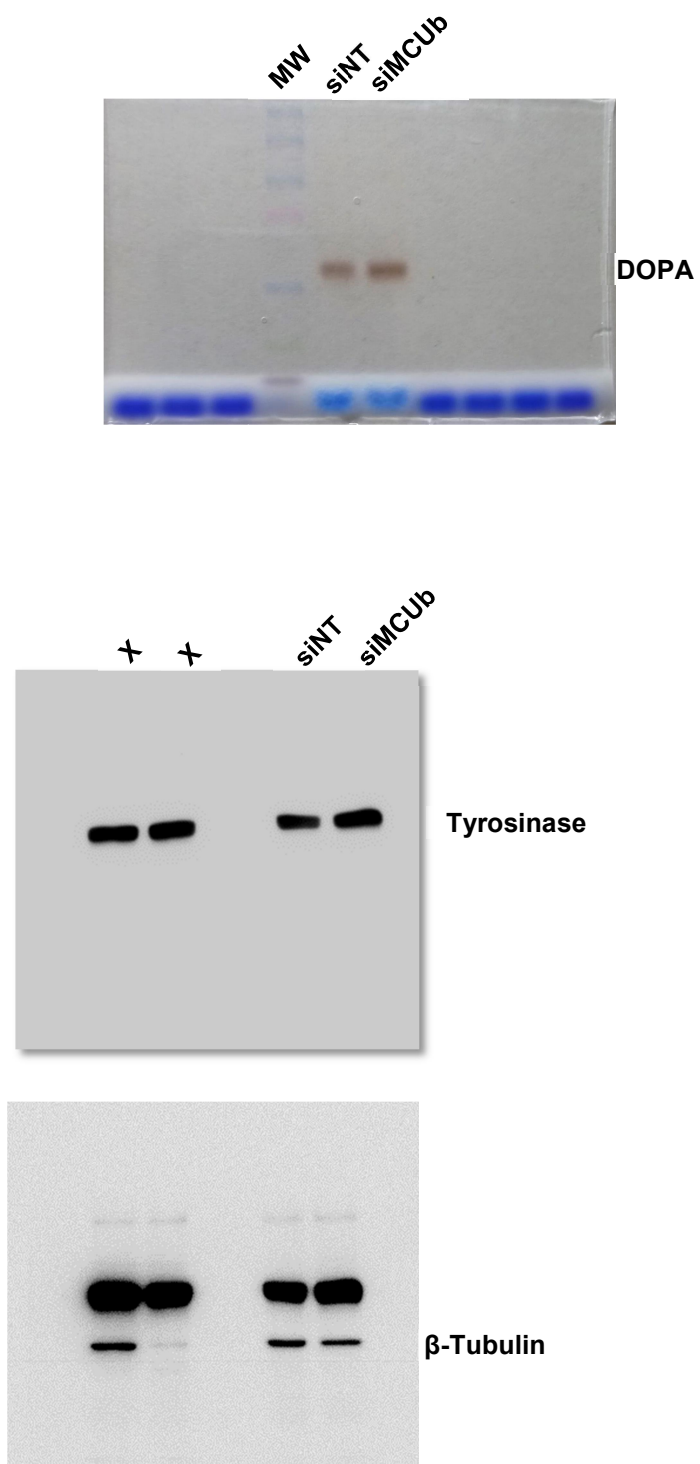

Supplement: S1 Raw Images — (PDF) [file pbio.3002895.s014.pdf]
