# Supplementary material for: Mitochondrial calcium uptake orchestrates vertebrate pigmentation via transcriptional regulation of keratin filaments
Source: PLoS Biol. 2024 Nov 11;22(11):e3002895. doi: 10.1371/journal.pbio.3002895 (PMC11581414; doi:10.1371/journal.pbio.3002895)
Supplement: Attachment — Submitted filename: Response to Reviewers Comments Draft 16 04062024.docx [file pbio.3002895.s015.docx]

**Editor and Reviewer Comments:     

Editors Comments:**

As you will see, the reviewers find the conclusions interesting and novel, however they also think the data doesn’t support some of the main conclusions and recommend several experiments to address this.

Both *Reviewers 1 and 2 are not convinced about the model and think it has some contradictions and shortcomings*. Thus, we do think it would be better to focus the manuscript on clearly demonstrating that mitochondrial Ca2+ uptake is playing a salient role in regulating melanogenesis.

Response: We really appreciate the feedback given by the Reviewers and the Editors. We have performed several additional experiments and have addressed all the concerns raised by the Reviewers. Further, as recommended, we have focused the manuscript on demonstrating that mitochondrial Ca^2+^ uptake plays a critical role in regulating melanogenesis. Accordingly, we have changed the title and modified abstract & text of the manuscript. We have also removed the summary figure with feedback loop from the manuscript. This revision has enhanced the quality of the manuscript. We hope that the Reviewers and the Editors will find the revised manuscript fit for acceptance.

1. Reviewer 2 also thinks that the mouse data are weak and incomplete because MCU knockouts have not been analysed, and that you should do so.

Response: We would like to thank Reviewer 2 and the Editor for this important comment. We have effectively addressed this comment and the new data provided in the revised manuscript corroborates our earlier results.

In the revised manuscript, we have analyzed pigmentation in both global MCU heterozygous knockout (MCU^+/-^) and global MCUb knockout (MCUb^-/-^) mice. These data demonstrates that MCU^+/-^ mice have significantly lower tail pigmentation whereas MCUb^-/-^ mice show markedly higher tail pigmentation (**Fig 3J and 3K** of the revised manuscript). We have validated corresponding changes in mitochondrial Ca^2+^ uptake in cells harvested from MCU^+/-^ and MCUb^-/-^ mice (**Fig 3G and 3I** of the revised manuscript). Since reviewers suggested that MCU knock-in mice is not well characterized model, we have replaced MCU knock-in mice data with MCU^+/-^ and MCUb^-/-^ mice data. We believe the new *in vivo* data, from these two independent transgenic mice models, has significantly enhanced the manuscript. Taken together, these data reveal a critical role for mitochondrial Ca^2+^ uptake in pigmentation.

Dr. Rosario Rizzuto’ lab has shared the MCU^+/-^ and MCUb^-/-^ transgenic mice data with us. Therefore, Dr. Rizzuto and his colleague are authors on the revised manuscript. Please find below mitochondrial Ca^2+^ imaging data from the cells isolated from MCU^+/-^ and MCUb^-/-^ transgenic mice, the mice tail pictures and quantitation of tail pigmentation:

**I.**

**H.**

**G.**

**K.**

**MCU^+/-^**

**WT**

**MCUb ^-/-^**

**J.**

2. Reviewer 3 points out that the fish data also needs additional experiments to confirm the main conclusions and that the overall effect should be demonstrated in at least one organism.

Response: We thank Reviewer 3 and the Editor for recommending that the overall effect of mitochondrial Ca^2+^ uptake should be demonstrated in at least one organism. As discussed above, in the revised manuscript, we have provided new pigmentation data from MCU^+/-^ and MCUb^-/-^ transgenic mice. Thereby confirming overall effect of mitochondrial Ca^2+^ signaling in the mammalian model system.

We would have loved to perform MCUb silencing and overexpression experiments in zebrafish as it is our core expertise. However, zebrafish lacks MCUb and in the course of evolution, MCUb appeared only after bony fishes (zebrafish is a bony fish) starting from amphibians such as xenopus (source: Genecards, ZFIN and Raffaello *et al.* EMBO J, 2013). Nonetheless, zebrafish possess EMRE and MICUs (MICU1, MICU2 and MICU3). MICUs via their gatekeeping activity must be regulating mitochondrial Ca^2+^ uptake in zebrafish. While during the course of evolution higher vertebrates have acquired MCUb as an additional strategy to tightly control mitochondrial Ca^2+^ uptake.

Please refer below to the phylogenetic analysis of MCUb:

3. Reviewer 4, along with Reviewer 2, mentions that you should demonstrate further that the key mechanism being proposed depends on NFAT regulation.

Response: In the revised manuscript, we have provided a series of new evidence that clearly demonstrates that NFAT plays a critical role in the proposed mechanism. We have performed following experiments to substantiate NFAT’s role:

a. As suggested by Reviewer 2 and Reviewer 4, we examined the effect of the calcineurin inhibitor cyclosporine A on αMSH induced melanogenesis. As shown in the **Fig 6K and 6L** of the revised manuscript, our dose response experiments suggest that cyclosporine A treatment at 10nM and 100nM does not alter αMSH stimulated pigmentation while at 10μM cyclosporine A inhibits αMSH induced pigmentation. Similar dose dependent effect of cyclosporine A in regulating pigmentation in primary human melanocytes was reported earlier (Lee et al. Pigment Cell Research 2003). Please refer to the responses to Reviewer 2 and Reviewer 4 for further details. In brief, our new data, earlier reports on primary melanocytes and clinical studies on patients clearly demonstrate that the effect of NFAT inhibitor is cell type, drug concentration and context dependent.

Please find below data from the cyclosporine experiments for your reference:

b. Secondly, as recommended by Reviewer 1, we performed additional luciferase assays with MyoD1 transcription factor (an additional control) and observed that co-expression of MyoD1 does not increase the luciferase activity of keratin5 promoter (**Fig 6H**). We further validated it by examining keratin5 mRNA levels upon MyoD1 overexpression and found that keratin5 levels are not altered (**Fig 6J** of the revised manuscript). Please refer to this new data below.

c. Finally, as suggested by Reviewer 3, we generated a mutant keratin5 promoter in which a highly conserved NFAT2 binding site (-924 to -913) was deleted. The luciferase assays with this mutant promoter show that upon co-expression of NFAT2 the luciferase activity of the mutant keratin5 promoter is significantly less in comparison to wild type keratin5 promoter thereby validating the regulation of keratin5 transcription by NFAT2 (**Fig 6H** of the revised manuscript). Please find below this additional data:

Collectively, this new data establishes that NFAT2 plays a crucial role in driving pigmentation by bridging changes in mitochondrial Ca^2+^ levels to keratin transcription. However, there might be other signaling modules working downstream of mitochondrial Ca^2+^ uptake to regulate pigmentation. But it is beyond the scope of this study to characterize all such pathways.

4. Reviewer 4 and Reviewer 1 also think that the Ca2+-imaging experiments need concomitant readouts of cytosolic Ca2+.

Response: We thank Reviewer 1, Reviewer 4 and the Editor for asking for this critical control experiment. As recommended by the Reviewers, we have performed concomitant cytosolic Ca^2+^ measurements in all the suggested experiments. These new data show that the enhanced mitochondrial Ca^2+^ uptake is neither due to increase in histamine receptors expression nor due to surge in ER Ca^2+^ release (**Fig 1E-F and 1M-N** of the revised manuscript). Thereby suggesting that the increased mitochondrial Ca^2+^ uptake is due to MCU complex mediated rise in Ca^2+^ levels within mitochondrial matrix.

Further, histamine and αMSH stimulated cytosolic Ca^2+^ measurements in MCU silencing, MCUb knockdown and MCU overexpression conditions demonstrate that αMSH stimulates MCU complex mediated mitochondrial Ca^2+^ dynamics just like histamine (**Fig 2C-F; Fig 2M-P and S2K-N Fig** of the revised manuscript). Taken together, this control data has substantially improved the quality of our manuscript.

5. It is crucial to demonstrate the phenomenon with MSH, a bonafide melanogenic signal. Please note that histamine-induced Ca2+ signals may not be relevant to the biology being explored here and can only be considered as ancillary data to MSH-induced Ca2+ signaling.

Response: We would like to thank the Reviewers and Editor for this highly relevant observation. As suggested by the Reviewers, we have performed a series of Ca^2+^ imaging experiments with αMSH. This new data reveal that αMSH can stimulate mitochondrial Ca^2+^ uptake. Further, αMSH induced mitochondrial uptake and concomitant changes in cytosolic Ca^2+^ upon MCU silencing, MCUb knockdown and MCU overexpression shows similar results as observed with histamine (**Fig 2D & 2F; Fig 2N & 2P and S2M-N Fig** of the revised manuscript). In summary, this additional data demonstrates that αMSH modulates MCU complex mediated mitochondrial Ca^2+^ dynamics just like histamine.

6. Finally, Reviewer 4 points out that mitochondrial Ca2+ imaging with Rhod2AM is prone to artifacts and that the current standard is to use a mitochondrially-targeted GECI such as CEPIA3mt.

Response: We agree with Reviewer 4 that Rhod2AM can give artifacts and that one should use mitochondrially-targeted GECI. Therefore, even in our original submission all mitochondrial Ca^2+^ imaging experiments, except one experiment in primary melanocytes, are performed with mitochondrially-targeted GECI CEPIA2mt. We are not able to carry out mitochondrially-targeted GECI based Ca^2+^ imaging in primary cells because DP melanocytes are extremely difficult to transfect. In last 10 years, we have tried several transfection reagents such as lipofectamine, dharmafect, turbofect, retroviruses and jetPRIME but we were not successful. Eventually, using nucleofection technology, we are able to transfect LP melanocytes but DP melanocytes could not survive after nucleofection. Please see below the pictures of LP and DP melanocytes one-day post nucleofection transfection:

Therefore, due to technical limitation, we are not able to perform one experiment with mitochondrially-targeted GECI. However, as an alternative strategy, we have included an additional control in the Rhod2AM imaging experiments. We co-stained primary melanocytes with Mito-tracker and have measured mitochondrial Ca^2+^ levels after normalizing it with the Mito-tracker signals. This strategy has significantly decreased the possibility of artifacts associated with Rhod2AM measurement. Similar strategy is adopted by other well established mitochondrial calcium Ca^2+^ groups as well (Pathak et al., eLife 2020; Dong et al. Mol Cell 2017 and Volonte et al. JBC 2024). In these additional experiments, we observe similar trend that the mitochondrial Ca^2+^ uptake is higher in DP melanocytes in comparison to LP melanocytes (**S1D Fig** of the revised manuscript and incorporated below). This new data further strengthens our original observation. We sincerely hope the Reviewer and editors would be satisfied with this additional data.

**Reviewer 1:**

Response: We thank the Reviewer for appreciating the novelty of our study and highlighting that this work would be of interest of mitochondrial community. Further, we would like to thank him/her for suggesting very important experiments. We have performed all the recommended experiments and this has significantly enhanced the quality of our manuscript.

**Specific comments:**

(1) Please use "photo" or "image" instead of "picture" while referring to the photos of the cell pellets.

Response: As suggested, we have replaced the word “picture” with “image” while referring to the photos of the cell pellets.

(2) Please explain how melanin content was measured and normalized, as opposed to referring to another paper. What is the melanin content normalized to? MCU knockdown can reduce cell growth (PMID: 31040260), did the authors correct for possibly lower cell number in these experiments? What was the zebrafish data normalized to? These are very important details to be able to conclude that MCU affects pigmentation, and not cell growth. What are the units on melanin graphs (ug/ 5lac cells)? What is 5lac? Please correct ug to μg where necessary.

Response: For the melanin content assay, an equal number of cells (say 5 lac or 0.5 million cells) are lysed by boiling them in 1N NaOH and the amount of melanin is estimated by measuring absorbance of the lysed cells at 405 nm. Absorbance is also calculated for different concentrations of synthetic melanin ranging from 0 to 150 μg/mL and these readings are used to make a standard curve to estimate melanin content from corresponding absorbance values. The raw melanin content values obtained from different lysates/samples are then interpolated on the standard curve to get the melanin content. Finally, melanin content is normalized to the respective control sample (siNT or empty vector) and is plotted as a single data point on the bar graphs. In case of zebrafish, the melanin content in MCU morphants and rescue condition was normalized to melanin content in control morphants. Since equal number of cells and fishes were used in control as well as experimental conditions, the possible contribution of differences in cell number is negated. Further, we have corrected ug to μg throughout the manuscript.

(3) Data presented in Figs. 1C, 1D, 1G are not enough to conclude that LD day6 or DP have higher mitochondrial calcium activity. The results could be an indication of increased histamine receptors, and increased calcium release from the ER, rather than increased mitochondrial calcium uptake activity. The authors should also monitor cytosolic calcium levels under these conditions at the very least.

Response: We thank the Reviewer for suggesting this important control experiment. As suggested, we have measured histamine induced changes in the cytosolic Ca^2+^ levels during different days of LD as well as in primary human melanocytes (LP and DP). Our data show that histamine induced cytosolic Ca^2+^ levels do not change significantly during LD pigmentation (LD day0, day4, day5 and day6). Further, similar measurements in LP and DP primary human melanocytes reveal that histamine induced cytosolic Ca^2+^ signals are comparable in LP and DP cells. If anything, the cytosolic Ca^2+^ signals are marginally (but not significantly) lower on LD day6 and in DP melanocytes in comparison to LD day0 and LP melanocytes respectively. Taken together, our live cell Ca^2+^ imaging data clearly demonstrate that there is no difference in cytosolic Ca^2+^ levels (both in LD and primary cells) upon histamine stimulation thereby ruling out the possibility of increased histamine receptors expression and/or enhanced ER Ca^2+^ release. This new data is included in the **Fig 1E-F and Fig 1M-N** of the revised manuscript.

(4) What happens to mitochondrial content during pigmentation? Is the increased MCU activity in LD day6 and DP cells due to more mitochondria? How specific is the MCU activity increase. Please add a western blot showing MCU levels, as well as 1-2 other mitochondrial protein markers. Otherwise, it is hard to conclude that MCU activity increases, and not mitochondrial content increases overall.

Response: We understand the Reviewer’s concern that the increase in mitochondrial content may lead to enhanced mitochondrial Ca^2+^ uptake during pigmentation. Therefore, we took a well-established and holistic approach of analyzing mitochondrial content using Mito-tracker dye. We performed confocal microscopy and our data clearly shows that the mitochondrial content remains unchanged during LD pigmentation (LD day0, day4 and day6) (**S1A and S1B Fig** of the revised manuscript). Further, similar experiments in LP and DP melanocytes corroborate that the mitochondrial content is comparable in primary LP and DP melanocytes (**S1E and S1F Fig** of the revised manuscript). This new data demonstrates that the overall mitochondrial content remains unchanged during pigmentation. Moreover, as suggested, we have provided western blotting data for MCU during pigmentation. The levels of MCU are higher on LD day6 and in DP melanocytes in comparison to LD day0 and LP melanocytes respectively (**Fig 1G-H and Fig 1O-P** of the revised manuscript). Collectively, this additional data suggests that during pigmentation mitochondrial content remains unchanged but MCU expression and activity is significantly augmented.

(5) In Fig 3G, please show the tails against the same background. The differences in pigmentation are not easy to see by eye, and the fact the one tail is on a darker background makes it hard to compare them. Both tails should be on a white background ideally.

Response: We agree with the Reviewer that the pigmentation differences are visually difficult to appreciate on different backgrounds. Therefore, as recommended, we have provided tail images on white background in the revised manuscript. Further, as suggested by the Editor and Reviewer 2, we have included mice pigmentation data from both MCU and MCUb knockout mice. These data demonstrates that MCU^+/-^ mice have significantly lower tail pigmentation whereas MCUb^-/-^ mice show distinctly higher tail pigmentation. The mice tail photos and their quantitation are included in **Fig 3J and 3K** of the revised manuscript.

(6) Figs. 5B and 5C show "representative" images, but without proper statistical analysis of mitochondrial calcium levels in response to Keratin 5 overexpression, it is not possible to conclude that Keratin 5 affects mitochondrial calcium levels. How many times was this experiment done, what is the noise in the experiment? Are these changes significant? The affect sized are so small, the claim that Keratin 5 regulates MCU function is not very convincing.

Response: We apologize to the Reviewer for not adequately highlighting the statistical analysis performed on mitochondrial Ca^2+^ uptake upon keratin5 overexpression and knockdown. Actually, the statistical analysis from over 100 cells in keratin5 overexpression and keratin5 silencing conditions were provided in the supplementary Fig 5 of the original submission (**S7A-D Fig** of the revised manuscript). This analysis shows that keratin5 overexpression significantly increases mitochondrial Ca^2+^ uptake while keratin5 silencing significantly decreases it. Collectively, the gain and loss of function studies reveal an important role of keratin5 in regulating mitochondrial Ca^2+^ dynamics.

(7) What are the red labelled nucleotides in Fig 6B? The authors claim that Keratin 5 promoter has consensus NFAT2 motifs (TTCC?), but their promoter alignment does not show this consensus. How good of a match are these sites, what metric is used? Please indicate any statistical evaluation/ score for NFAT2 consensus sequences in Keratin 5 promoter.

Response: We apologize to the Reviewer for not being very clear in the original promoter alignment. In the revised manuscript, we have reorganized the alignment to better demonstrate NFAT2 binding sites (**Fig 6B**). The NFAT2 consensus sites are now labelled in red. The labelled NFAT2 binding sites have been validated by 2 independent DNA binding analysis software i.e. EPD Search Motif Tool and ContraV3. Only the sites obtained from both software are identified as potential NFAT2 binding sites. The EPD data was used at a p-value cutoff of 0.01 and the ContraV3 data was characterized at the high stringency setting with core similarity score of 0.95 and similarity matrix score of 0.85.

(8) The authors lack proper controls for the experiments shown in Figs. G and H. Would co-expression of any transcription factor with their lacZ reporter cause an increase? How specific is this signal. Empty vector is not good control, the authors should use another TFs and convince the reader that the increased LacZ signal they observe is specific it NFAT2, and is not observed due to overexpression of any transcription factor.

Response: As recommended by the Reviewer, we performed additional luciferase assays with MyoD1 (a muscle specific transcription factor) and observed that co-expression of MyoD1 does not increase the luciferase activity of keratin5 promoter (**Fig 7H** of the revised manuscript). We further validated it by examining keratin5 mRNA levels upon MyoD1 overexpression and found that keratin5 levels are not altered (**Fig 7J** of the revised manuscript). Moreover, as suggested by Reviewer 3, we generated a mutant keratin5 promoter in which a highly conserved NFAT2 binding site was deleted. The luciferase assays with this mutant promoter show that upon co-expression of NFAT2 the luciferase activity of the mutant keratin5 promoter is significantly reduced in comparison wild type keratin5 promoter thereby validating the regulation of keratin5 transcription by NFAT2 (**Fig 7H** of the revised manuscript). Taken together, this new data demonstrates that NFAT2 drives keratin5 promoter activity.

**Reviewer 2:**

Response: We thank the Reviewer for giving critical feedback on our study. In the revised manuscript, we have performed experiments recommended by the Reviewer and this has substantially improved the quality of our work.

**MAJOR CONCERNS:**

1. MCU's role in mammalian skin pigmentation seems very modest. In B16 cells, the knockdown of MCU results in an <20% decrease in melanin production (Fig. 2F). In primary melanocytes, MCU knockdown also produced a very modest effect (n=1, no quantification, Fig S1K). Authors show increased pigmentation in the tails of gene-edited mice but the C97A mutation has not been adequately characterized through electrophysiology of mitoplasts and it's not clear if melanocytes derived from these mice indeed show substantially increased mitochondrial Ca2+ uptake. Also note that the quantification of this data is not in accord with best practice - the sample size has been made deceptively high by conflating multiple measurements from each mouse tail, with an actual total of only 3 mice (Fig. 3H). The statistical significance of such analyses cannot be taken too seriously - I think authors should use average readings from each mouse tail as a single datapoint and increase the sample size
of the mice to >7 for optimal statistical power. Note that hyperpigmentation is not at all obvious in the representative image (Fig. 3G). Pointing out these weak data may seem like nitpicking, but this central claim of the manuscript has been made without the most direct experiment for such a conclusion. The crucial experiment that would establish this conclusively is the demonstration of pigmentation defects in Melanocyte-specific knockout mice of MCU. Note that MCU global knockouts are viable in some backgrounds, and no one has reported any perceptible pigmentation defects in these mice. The data presented in this manuscript did not convince me that MCU plays an especially salient role in mammalian skin pigmentation. I found the data on fly pigmentation convincing, but all further analyses are on mammalian skin pigmentation.

Response: We thank the Reviewer for finding the zebrafish pigmentation data convincing. Regarding mice data, as suggested by the Reviewer, we have analyzed data from 7 mice/condition from two independent transgenic (MCU^+/-^ and MCUb^-/-^) mice (**Fig 3J** of the revised manuscript). These new data show that MCU^+/-^ mice have significantly lower tail pigmentation whereas MCUb^-/-^ mice demonstrate distinctly higher tail pigmentation (**Fig 3J and 3K** of the revised manuscript). Further, we have validated equivalent changes in the mitochondrial Ca^2+^ uptake in the cells harvested from MCU^+/-^ and MCUb^-/-^ mice (**Fig 3G-I** of the revised manuscript). Collectively, this data validates a critical role of mitochondrial Ca^2+^ uptake in mammalian skin pigmentation as well. The possible reasons why other groups working with MCU global knockouts have missed observing changes in the pigmentation could be:

a. The viable MCU global knockouts mice have been reported to acquire compensatory mechanisms to survive in absence of MCU (Harrington and Murphy J Mol Cell Cardiol 2015; Murphy et al. BBRC 2014). Furthermore, the global knockouts have been demonstrated to show cell specific compensatory outcomes when compared with transient MCU silencing and/or conditional tissue specific knockouts in adult mice (Luongo et al. Cell Reports 2015; Kwong et ai. Cell Reports 2015; Nichols et al. Journal of Cerebral Blood Flow and Metabolism. 2017; Nichols et al. Cell Death and Disease 2018; Wu et al. Front. Pharmacol. 2021; Qin et al. Experimental Neurology 2023 ). Therefore, it may be possible that certain feedback/compensatory mechanisms in MCU^-/-^ mice rescue pigmentation phenotype that we have observed in MCU^+/-^ mice.

b. Secondly, it is very easy to observe changes in the follicular (fur) pigmentation in mice which is equivalent to human hair pigmentation while people may miss out on recognizing the changes in epidermal (tail) pigmentation until and unless they pay critical attention to tail colors across different genotypes of mice.

Further, we have provided quantitation of MCU knockdown phenotype in primary human cells (**S3I Fig** of the revised manuscript). We agree with the Reviewer that in B16 and primary melanocytes silencing of MCU results in 20-25% decrease in pigmentation but it is highly consistent. Further, we see around 50% increase in pigmentation with MCU overexpression while with MCUb knockdown we observe >65% increase in pigmentation. Collectively, the *in vitro* data shows a significant role of mitochondrial Ca^2+^ uptake in pigmentation. It is important to highlight that even silencing of master transcriptional regulator of pigmentation i.e. MITF results in only around 50% decrease in pigmentation in similar cellular pigmentation assays (Motiani et al. EMBO J 2018 and Tanwar et al. JBC 2022). Most importantly, our *in vivo* data from two distinct organisms (zebrafish and mice) including two independent transgenic mice models (MCU^+/-^ and MCUb^-/-^) demonstrate a critical role of MCU complex mediated mitochondrial Ca^2+^ uptake in pigmentation.

2. Authors have previously reported that in the B16 cell line MSH-induced pigmentation depends on Ca2+ signaling and that MSH triggers the release of ER Ca2+. But in this paper, all the measurements of mitochondrial Ca2+ are in response to Histamine, which is perplexing because to the best of my understanding, Histamine is not a melanogenic stimulus. This seems conceptually disconnected because it would be important to know if MSH stimulates the uptake of mitochondrial Ca2+ and how this might affect cytosolic Ca2+ signals. The differences seen in response to Histamine, e.g. Fig. 1C, could simply be because the cells upregulate the Histamine receptors during the 6 days of MSH-induced pigmentation protocol. Crucially, authors do not show corresponding cytosolic Ca2+ signals - this is problematic. This same issue also applies to: (1) differences in primary melanocytes of Caucasian and African origin (Fig. 1G-H); (2) analyses of MCU and MCUb knockdown and overexpression in Fig.3.

Response: We thank the Reviewer for this important comment. Reviewer 1 has also asked similar question on the potential contribution of increased histamine receptors expression and/or enhanced ER Ca^2+^ release to histamine stimulated increase in mitochondrial Ca^2+^ uptake during LD pigmentation assay and in darkly pigmented (DP) human melanocytes. Therefore, as suggested, we have measured histamine induced changes in the cytosolic Ca^2+^ levels during different days of LD as well as in primary human melanocytes (LP and DP). Our data show that cytosolic Ca^2+^ levels do not change during LD pigmentation (LD day0, day4, day5 and day6) (**Fig 1E and 1F** of the revised manuscript). Further, similar measurements in LP and DP primary human melanocytes reveal that cytosolic Ca^2+^ levels are comparable in LP and DP cells (**Fig 1M and 1N** of the revised manuscript). Taken together, our live cell Ca^2+^ imaging data clearly demonstrate that there is no difference in cytosolic Ca^2+^ levels (both in LD and primary cells) upon histamine stimulation.

Further, as recommended, we have performed histamine stimulated cytosolic Ca^2+^ measurements upon MCU and MCUb knockdown as well as MCU overexpression. As expected MCU silencing increases histamine induced rise in cytosolic Ca^2+^ levels (**S2E and S2F Fig** of the revised manuscript) whereas MCUb silencing decreases it (**S4C and S4D Fig** of the revised manuscript) in comparison to control siRNA transfected cells. Similarly, MCU overexpression leads to reduction in histamine stimulate rise in cytosolic Ca^2+^ levels as compared to empty vector control **S3A and S3B Fig** of the revised manuscript). Taken together, this additional data from several experimental conditions elegantly show that the differences in mitochondrial Ca^2+^ uptake presented in the original submission are neither due to increase in histamine receptors expression nor due to enhanced ER Ca^2+^ release.

Moreover, we performed αMSH induced mitochondrial Ca^2+^ uptake assays to reveal that αMSH can stimulate mitochondrial Ca^2+^ uptake. Importantly, in the revised manuscript, we have provided αMSH stimulated mitochondrial Ca^2+^ imaging data along with corresponding cytosolic Ca^2+^ measurements in MCU (**Fig 2D & 2F and S2G & S2H Fig** of the revised manuscript) and MCUb (**Fig 2N & 2P and S4E & S4F Fig** of the revised manuscript) knockdown as well as MCU overexpression conditions (**S2M & S2N Fig** **and** **S3C Fig** of the revised manuscript). The αMSH induced mitochondrial Ca^2+^ uptake data shows same results as seen with histamine stimulated mitochondrial Ca^2+^ uptake. In summary, our additional data demonstrates that αMSH modulates MCU complex mediated mitochondrial Ca^2+^ dynamics just like histamine.

3. Authors have proposed a mechanistic model wherein mitochondria Ca2+ uptake activates NFAT2. How does that happen? Activation of NFAT2 requires a sustained increase in cytosolic Ca2+ and downstream activation of calcineurin (which dephosphorylates NFAT). But authors have not dissected these aspects at all. First, NFAT2 translocation needs to be shown in response to MSH (not Histamine). Second, if NFAT2 activation is indeed crucial for melanogenesis, then cyclosporine, a highly potent nanomolar inhibitor of calcineurin should prevent MSH-induced pigmentation when used at 10 nM. However, it should be noted that cyclosporine is a widely used drug in transplant patients and despite long-term treatment with cyclosporine, these patients do not show any loss of pigmentation. In fact, there are case reports of hyper-pigmentation in patients taking cyclosporine. These issues again lead me to believe that the claim of MCU in mammalian skin pigmentation may have been overstated.

Response: We agree with the Reviewer that activation of NFAT2 requires an increase in cytosolic Ca^2+^ signals. Therefore, we examined both histamine and αMSH induced changes in cytosolic Ca^2+^ levels upon MCU silencing, MCU overexpression and MCUb knockdown. As expected, we observed that upon MCU silencing cytosolic Ca^2+^ levels were elevated (**S2E-H Fig** of the revised manuscript) whereas upon MCU overexpression and MCUb knockdown cytosolic Ca^2+^ levels were decreased (**S3A-C Fig** and **S4C-F Fig** of the revised manuscript). This data show that αMSH modulates MCU complex mediated mitochondrial and cytosolic Ca^2+^ dynamics just like histamine. It is important to highlight that similar results have been shown in other cellular models wherein inhibition of mitochondrial Ca^2+^ uptake results in elevation of cytosolic Ca^2+^ levels and activation of Ca^2+^ regulated transcription factors such as NFAT1/4 (REF: Yoast et al. JBC 2021) and NF-𝜅B (REF: Seegren et al. Nature Aging 2023). In brief, just like other cell models, perturbation in mitochondrial Ca^2+^ uptake in melanocytes results in increased cytosolic Ca^2+^ levels and activation of NFAT2.

Further, as suggested by the Reviewer, we investigated the role of cyclosporine A in regulating αMSH induced pigmentation. Literature survey suggests that cyclosporine A is used at various concentrations across different cell types. Therefore, we performed dose response assays with cyclosporine A. Our dose dependent experiments suggest that cyclosporine treatment at 10nM to 100nM concentrations does not alter αMSH stimulated pigmentation while at 10μM concentration cyclosporine inhibits αMSH induced pigmentation (**Fig 6K and 6L** of the revised manuscript). Similar dose dependent effect of cyclosporine A in regulating pigmentation in primary human melanocytes is already reported (Lee et al. Pigment Cell Research 2003).

As pointed out by the Reviewer, cyclosporine A is clinically used as an immunosuppressant. Literature survey to examine the effect of cyclosporine A intake on pigmentation suggests that it can induce pigmentation by acting on immune cells. It is important to highlight that immune cell activation is associated with hypopigmentation and indeed, vitiligo (de-pigmentary disorder) is an outcome of enhanced auto-immunity. Therefore, systemic administration of cyclosporine A might be showing pigmentary effects via cyclosporine A’s action on immune cells and it might not be directly associated with cyclosporine A’s action on melanocytes. Actually, the discrepancy in the action of cyclosporine A on immune cells and melanocytes has been reported earlier (Lee et al. Pigment Cell Research 2003). It was shown that cyclosporine treatment on primary human melanocytes results in decrease in pigmentation and tyrosinase activity (Lee et al. Pigment Cell Research 2003). In summary, our new data, earlier reports on primary melanocytes and clinical studies on patients clearly demonstrate that the effect of cyclosporine A is cell type, drug concentration and context dependent.

4. If one supposes that mitochondrial Ca2+ is a significant regulator of pigmentation, then the underlying mechanism proposed by the authors seems incoherent because there is no direct link to pigmentation machinery. Authors show some data suggesting that K5 regulates MCU, but this does not really address the key issue - how does mitochondrial Ca2+ uptake affect melanogenesis? The feedback model discussed by the authors is very confusing and does not add much to the manuscript. Perhaps there are other transcription factors that are activated by increase in cytosolic Ca2+ when mitochondria do not buffer the Ca2+ but this has not been explored.

Response: We understand the Reviewer’s concern but we do not agree that “there is no link of mitochondrial Ca^2+^ uptake to pigmentation machinery”. The data presented in **Fig 2, Fig 5, S3 Fig and S4** **Fig** show that mitochondrial Ca^2+^ uptake regulates expression of key melanosome structural protein Gp100 and critical enzymes involved in melanin synthesis i.e. Tyrosinase & DCT. Importantly, the changes in the melanosome structural protein and melanin synthesis enzymes modulate melanosome biogenesis and maturation (**Fig 5J-M**).

As discussed in response to comment 1 of the Reviewer, it is well appreciated that cells activate compensatory mechanisms to minimize the effects of decrease in MCU expression and we think that keratin mediated increase in mitochondrial Ca^2+^ uptake could be one such mechanism. However, we completely agree with the Reviewer that apart from this signaling module, there might be other transcription factors and/or signaling modules that could contribute to pigmentation downstream of mitochondrial Ca^2+^ uptake. Indeed, in the transcriptomics analysis, we observed seven signaling pathways that are differentially regulated upon MCU and MCUb silencing. We selected keratins based on their high enrichment in this analysis and their association with human pigmentary disorders. As Reviewer would agree, it is beyond the scope of this manuscript to characterize all other differentially regulated signaling pathways.

MINOR CONCERNS

5. The implicit claim is that during melanogenesis, the mitochondrial Ca2+ uptake machinery is activated or enhanced but the use of CEPIA and Rhod-2AM also integrates upstream changes in the mobilization of cytosolic Ca2+. Quantification of mitochondrial Ca2+ uptake capacity in isolated mitochondria or digitonin permeabilized cells will get around that but authors have not carried out these studies. Why not include these direct measurements?

Response: We agree with the Reviewer that upstream changes in the mobilization of cytosolic Ca^2+^ can affect mitochondrial Ca^2+^ uptake. Indeed, similar observation was made by Reviewer 1, Reviewer 4 and the Editor. Therefore, to rule out the potential contribution of differences in mobilization of cytosolic Ca^2+^, we performed cytosolic Ca^2+^ measurements both during LD pigmentation and in primary human melanocytes (lightly and darkly pigmented). These new data demonstrate that cytosolic Ca^2+^ does not change during melanogenesis (**Fig 1E & 1F and Fig 1M & 1N**). Thereby suggesting that the increased mitochondrial Ca^2+^ uptake is due to MCU complex mediated rise in Ca^2+^ uptake.

6. In a related aspect, please show the time lapse images of mitochondrial Ca2+ uptake in response to aMSH and Histamine (using CEPIA and Rhod2AM). These can be in the supplementary figures and the entire series need not be shown.

Response: As recommended by the Reviewer, we have included time lapse images of mitochondrial Ca^2+^ uptake in response to αMSH and histamine in the **S9 Fig and S10 Fig** of the revised manuscript.

7. The effect of mitoxantrone on MSH-induced mitochondrial uptake and cytosolic Ca2+ signals should be shown in melanocytes. This drug was reported to inhibit MCU >5y ago but many investigators have failed to replicate these findings and currently, there is not a consensus that it inhibits MCU. Also, it would be good to check if there are credible case reports of Mitoxantrone resulting in loss of pigmentation in MS patients who take it for years. I could not find any.

Response: As suggested by the Reviewer, we examined effect of mitoxantrone on αMSH induced mitochondrial Ca^2+^ uptake and cytosolic Ca^2+^ levels. In our experiments, we observe a significant decrease in αMSH stimulated mitochondrial Ca^2+^ uptake where cytosolic Ca^2+^ levels increase. This suggests that in melanocytes, mitoxantrone inhibits αMSH induced mitochondrial Ca^2+^ uptake. The new Ca^2+^ imaging data is included in **Fig 7A-D** of the revised manuscript.

Further, we surveyed the literature for case reports on mitoxantrone leading to loss of pigmentation in MS patients but we were not able to find reliable reports. A possible explanation could be that mitoxantrone is administrated by intravenous route and the concentration of mitoxantrone reaching melanocytes (localized in poorly vascularized epidermis layer) may not be sufficient to inhibit MCU and decrease pigmentation.

**Reviewer 3:**

Response: We thank the Reviewer for appreciating our study and suggesting experiments to further enhance the quality of our work.

1) What are the origin of B16 cells? Caucasian? and how they compare to a primary cell in term of pigmentation? This information in critical to results interpretation

Response: B16 cells are of mouse origin. B16 is one of the most commonly used cell lines in pigmentation biology. B16 cells are non-pigmented under resting conditions but they respond to melanogenic stimuli and pigment; thereby they serve as an excellent model system to study the pigmentation process. On the other hand, primary melanocytes are already pigmented and therefore, they can be only used to study changes in the pigmentation levels. In short, both B16 and primary cells have their strengthens and weaknesses as a pigmentation model system. Therefore, in this study, we have used both B16 and primary cells to demonstrate the role of mitochondrial Ca^2+^ uptake in pigmentation. Further, we have corroborated our *in vitro* data in two independent model organisms.

2) Are the primary human melanocytes (Fig 1E)also seeded at low density? If so, what happens when you perform a similar time course assay as in Fig 1 A-B

Response: Since primary human melanocytes are already pigmented, they are not seeded at low density to induce pigmentation.

3) Fig 2. Does MCU overexpression leads to increase in mitochondrial calcium uptake? This is important. If this is the case, then it is quite curious that excess overexperessed MCU proteins would be functional without accessory molecules such as EMRE and other proteins part of the complex? Are these proteins upregulated when MCU is overexpressed?

Response: To address this comment, we measured histamine and αMSH induced mitochondrial Ca^2+^ uptake as well as corresponding changes in cytosolic Ca^2+^ levels upon MCU overexpression. Our data show that ectopic expression of MCU increases mitochondrial Ca^2+^ uptake and decreases cytosolic Ca^2+^ levels. This new data is presented in the **S2K-N Fig** and **S3A-C Fig** of the revised manuscript. Similar data is already reported in the MCU discovery paper i.e. MCU overexpression results in enhanced histamine induced mitochondrial Ca^2+^ uptake while cytosolic Ca^2+^ level is decreased (De Stefani et al. Nature 2010).

Further, as suggested by the Reviewer, we examined the levels of other MCU complex member i.e. MCUb, EMRE, MICU1 and MICU2 upon MCU overexpression. Our data suggests that MCU overexpression does not change the expression of other components of the MCU complex (**S3D Fig**).

4) Data in Supplementary Fig S1F should be address at the same time as Fig 1. Or these data should move to Fig 1. How about other proteins of the MCU complex, are they also highly expressed in DP vs LP?

Response: As recommended by the Reviewer, we have moved MCU expression data to **Fig 1O and 1P** of the revised manuscript. Further, we analyzed levels of other proteins of MCU complex i.e. MCUb, EMRE, MICU1 and MICU2 both during LD pigmentation and in primary melanocytes. Our qRT-PCRs data suggests that in general the expression of MCU complex proteins is increased during LD pigmentation (**S1C** **Fig** of the revised manuscript). Moreover, their expression is higher in DP melanocytes in comparison to LP melanocytes (**S1G** **Fig** of the revised manuscript).

5) Re: DP vs LP: I think it would be more judicious to silence MCU in the DP melanocytes to see if this will affect pigmentation?

Response: In the revised manuscript, we have provided MCU silencing data in several independent model systems i.e. B16 cells, primary LP melanocytes, zebrafish and transgenic MCU^+/-^ mice. We agree with the Reviewer that along with these models, it would be good to knockdown MCU in DP melanocytes as well. However, due to the technical difficulty in performing transfections in DP melanocytes (please refer to the response to a similar comment from the Editor for details), we could not carry out MCU silencing in DP melanocytes. We believe that the Reviewer would appreciate that the data presented in the revised manuscript clearly demonstrates a critical role of MCU in pigmentation.

6) Along the same lines, Are the levels of MCUb different between LP and DP melanocytes? This is also important!

Response: As suggested by the Reviewer, we analyzed MCUb levels in LP and DP melanocytes by performing qRT-PCRs. Our data suggests that MCUb expression is higher in DP melanocytes as compared to LP melanocytes. This data is presented in **S1G Fig** of the revised manuscript.

7) The authors should also silence and/or overexpress MCUb in DP melanocytes.

Response: As discussed above and explained in detail in response to the Editor’s comment, it is technically challenging to perform transfections in DP melanocytes. However, in the revised manuscript, we have provided data from transgenic MCUb^-/-^ mice that elegantly reveals an important role of MCUb in pigmentation. The tail pigmentation is significantly higher in MCUb^-/-^ mice in comparison to wild type control mice. Taken together, our data demonstrates that MCUb negatively regulates pigmentation.

8) Did the authors inject MCUb RNA in Zebrafish? These in principle should reduce melanogenesis. What about MCUb morpholinos?

Response: We thank the Reviewer for suggesting this relevant question. As discussed in response to a similar comment from Editor, zebrafish lacks MCUb and it is logically not possible to perform MCUb silencing and overexpression studies in zebrafish. To strengthen our *in vivo* data with MCUb, we analyzed tail pigmentation in MCUb^-/-^ transgenic mice. In the revised manuscript, we have provided new pigmentation data from MCUb^-/-^ mice in **Fig 3J and 3K**. Taken together, we have validated a critical role of MCUb in pigmentation *in vivo* by utilizing a transgenic mice model.

9) Mice KI MCU: Show additional tails to enable visual comparisons by the reader. Show data that indeed KI cells have increased Mitochondrial Ca2+ uptake or high mitochondrial calcium at the basal level.

Response: We thank the Reviewer for this important comment. Reviewer 1 has also asked similar question on the mice tail photos and Reviewer 2 suggested to show tail images from MCU knockout mice. Therefore, in the revised manuscript, we have shown 3 tail images each from MCU^+/-^ and MCUb^-/-^ mice along with control wild type mice on white background (**Fig 3J** of the revised manuscript). Further, we quantitated tail pigmentation in 7 mice/condition (**Fig 3K** of the revised manuscript). Moreover, we have corroborated analogous changes in the mitochondrial Ca^2+^ uptake in the cells harvested from MCU^+/-^ and MCUb^-/-^ mice (**Fig 3G-I** of the revised manuscript). Taken together, this data reveal a critical role of mitochondrial Ca^2+^ uptake in pigmentation.

10) Fig 5 I: these blots are not very convincing. Please show better quality blots. Please make sure background are visible in all blots. Please provide full uncut and unprocessed blots related to all data.

Response: As suggested by the Reviewer, we have provided better quality blots for the Fig 5I of the original submission (**Fig 5I** of the revised manuscript). Further, we have included full uncut and unprocessed blots in S1 Raw Images PDF file.

11) Fig 6: Authors should mutate this NFAT2 putative promoter region and determine if luciferase activity is reduced

Response: As suggested by the Reviewer, we generated a mutant keratin5 promoter in which a highly conserved NFAT2 binding site (-924 to -913) was deleted. Our new data show that NFAT2 driven luciferase activity of the mutant keratin5 promoter is significantly reduced in comparison to wild type keratin5 promoter (**Fig 6H** of the revised manuscript). This additional data further substantiates that NFAT2 drives keratin5 promoter activity**.**

**Reviewer 4:**

Response: We thank the Reviewer for appreciating our paper and highlighting the importance of biological question that we have addressed via this study.

**Major:**

1. The study appears oversimplified, lacking crucial controls. The authors overlook the role of SOCE as well as other mitochondrial parameters influenced by MCU, potentially involved in pigmentation control.

Response: We thank the Reviewer for pointing out to lack of few controls. In the revised manuscript, we have performed several additional controls such as αMSH induced mitochondrial Ca^2+^ uptake and corresponding cytosolic Ca^2+^ measurements; analysis of histamine stimulated cytosolic Ca^2+^ signals during LD pigmentation and in primary human melanocytes; and mitochondrial Ca^2+^ uptake assays in the cells harvested from MCU^+/-^ and MCUb^-/-^ mice. Collectively, these experiments have strengthen our original data.

2. Previous research on MCU, SOCE, and NFAT interplay is mainly disregarded. Given SOCE's known role in melanin synthesis and MCU's control over SOCE, further investigation into the MCU-SOCE-NFAT interplay is needed.

Response: We agree with the Reviewer that SOCE can regulate melanogenesis and MCU controls SOCE. Hence, there is a possibility that MCU could regulate melanogenesis via SOCE-NFAT interplay. However, we unbiasedly characterized the molecular pathways working downstream of MCU to modulate pigmentation by performing transcriptomics analysis and narrowed down to keratins. Further, it would take substantial time and resources to systematically and precisely investigate the role of MCU-SOCE-NFAT axis in pigmentation. Therefore, we believe that it is beyond the scope of this manuscript to delineate this signaling module. We sincerely hope that the Reviewer would be satisfied with the numerous additional experiments performed to revise this manuscript.

3. The impact of genetic manipulations of MCU, MCUb, and keratin 5 on mitochondrial function and bioenergetics is not addressed.

Response: It is correct that we have not studied effect of MCU, MCUb and keratin5 perturbations on all mitochondrial functions like mitochondrial dynamics, bioenergetics, changes in metabolic fluxes etc. However, we have provided significant amount of novel data that demonstrates an important role of MCU, MCUb and keratin5 in melanogenesis. In this study, we have focused on mitochondrial Ca^2+^ signaling and have performed a variety of experiments in distinct model systems to reveal its key role in vertebrate pigmentation. We understand that it is important to examine the role of MCU, MCUb and keratin5 in other mitochondrial functions. However, we think that it is beyond the scope of this manuscript to cover other mitochondrial functions. We sincerely hope that the Reviewer would agree that it would be better to carry out detailed investigation to delineate the significance of MCU, MCUb and keratin5 in other mitochondrial function as an independent study.

4. The role of SOCE, NFAT and MCU have been investigated in melanoma cells previously. Was there any effect on cell pigmentation reported? Does treatment of melanoma cells but also patients with calcineurin (and hence NFAT) inhibitors (tacrolimus etc.) affect their pigmentation?

Response: The role of SOCE players STIM and Orai proteins is reported in both melanoma and pigmentation (Stanisz et al. J Invest Dermatol 2012; Stanisz et al. PCMR 2014; Hooper et al. Mol Cell Biol 2015; Stanisz et al. J Physiol 2016; Motiani et al. EMBO J 2018 and Gross et al. EMBO J 2022). The work is majorly led by Bogeski and Soboloff groups. Further, Bogeski lab has reported role of SOCE in both melanoma and pigmentation but in independent studies. In one of our earlier studies, we investigated the role of STIM1 in both melanoma and pigmentation. We demonstrated that STIM1 can regulate not only melanoma progression but also melanogenesis (Motiani et al. EMBO J 2018). However, it is important to highlight that the assays, which are performed for analyzing melanogenesis versus melanoma progression are very different. Therefore, until the appropriate experiments are conducted, it might be very difficult to reveal the role of any protein in pigmentation.

The calcineurin inhibitors (cyclosporine A and tacrolimus) are clinically used as immunosuppressants. Literature survey to examine their role in pigmentation suggests that they can induce pigmentation by acting on immune cells. It is important to highlight that immune cell activation is associated with hypopigmentation and actually, vitiligo (de-pigmentary disorder) is an outcome of enhanced auto-immunity. Therefore, systemic administration of calcineurin inhibitors might be showing pigmentary effects via their action on immune cells and it might not be directly associated with their action on melanocytes. Indeed, the discrepancy in the action of cyclosporine A on immune cells and melanocytes is already reported (Lee et al. Pigment Cell Research 2003). It was shown that cyclosporine treatment on primary human melanocytes results in decrease in pigmentation and tyrosinase activity (Lee et al. Pigment Cell Research 2003). Further, the dose response experiments suggest that cyclosporine A treatment at 10nM to 100nM concentrations does not alter αMSH stimulated pigmentation while at 10μM concentration cyclosporine A inhibits αMSH induced pigmentation (**Fig 6K and 6L** of the revised manuscript). In summary, our new data, earlier reports on primary melanocytes and patients demonstrate that the effect of calcineurin inhibitors is cell type, drug concentration and context dependent.

**Minor:**

1. Using murine melanoma cells for pigmentation studies is not the most appropriate approach.

Response: We agree with the Reviewer that B16 cells are not the most appropriate model system to study pigmentation. Still, B16 is one of the most commonly used cell lines in pigmentation biology. B16 cells are non-pigmented under resting conditions and pigment in response to melanogenic stimuli thereby they provide a good model system to study the pigmentation process. On the other hand, primary melanocytes are already pigmented and therefore, they can be only used to study changes in the pigmentation levels. In short, both B16 and primary cells have their pros and cons as a pigmentation model system. Therefore, in this study, we have used both B16 and primary human melanocytes to show the role of mitochondrial Ca^2+^ uptake and keratin5 in pigmentation. Further, we have corroborated our *in vitro* data in two independent model organisms i.e. zebrafish and mice.

2. Given that alphaMSH causes calcium uptake (published by the same authors) the use of artificial stimulus such as histamine needs to be explained.

Response: We thank the Reviewer for pointing out to the use of histamine in mitochondrial Ca^2+^ uptake assays. In the revised manuscript, we have provided new data with αMSH induced mitochondrial Ca^2+^ uptake. The data with αMSH essentially corroborates the mitochondrial Ca^2+^ uptake data obtained with histamine. We had originally used histamine in our study as the signals are more robust with histamine (85-90% cells respond to histamine) while with αMSH 55-60% cells respond, which is similar to the proportion of cells that pigment upon αMSH treatment.

3. Rhod2AM is prone to artifacts. The authors should use CEPIA or similar for all measurements of mCa2+.

Response: We agree with Reviewer 4 that Rhod2AM can give artifacts and that one should use mitochondrially-targeted GECI. Therefore, in our original submission all mitochondrial Ca^2+^ imaging experiments, except one experiment in primary melanocytes, were performed with mitochondrially-targeted GECI CEPIA2mt. We are not able to carry out mitochondrially-targeted GECI based Ca^2+^ imaging in primary cells because DP melanocytes are extremely difficult to transfect. Please refer to the response to a similar comment from Editor for details.

However, as an alternative strategy, we have included an additional control in the Rhod2AM imaging experiments. We co-stained primary melanocytes with Mito-tracker and have measured mitochondrial Ca^2+^ levels after normalizing it with the Mito-tracker signals. In these additional experiments, we observe similar trend that the mitochondrial Ca^2+^ uptake is higher in DP melanocytes in comparison to LP melanocytes (**S1D Fig** of the revised manuscript). This new data further strengthens our original observation. We sincerely hope the Reviewer would be satisfied with this new data.

4. How many melanocyte lines were used for the data shown in Fig. 1 E-H? Only 1+1?

Response: We performed four independent biologicals of mitochondrial Ca^2+^ imaging experiments in primary melanocytes arising from three distinct lots of cells. Therefore, at least 3 distinct LP and DP lines were used in performing these experiments. These distinct DP and LP lines include the ones used for generating additional data, during the revision, with Mito tracker green and Rhod 2AM co-staining.

5. Is the MCU expression increased in the samples shown in Fig 1A?

Response: Yes, MCU expression is increased on LD day6 in comparison to LD day0. This new data is provided in the **Fig 1G and 1H** of the revised manuscript.
