# Supplementary material for: Mitochondrial calcium uptake orchestrates vertebrate pigmentation via transcriptional regulation of keratin filaments
Source: PLoS Biol. 2024 Nov 11;22(11):e3002895. doi: 10.1371/journal.pbio.3002895 (PMC11581414; doi:10.1371/journal.pbio.3002895)
Supplement: Attachment — Submitted filename: Response to Reviewers Comments Draft 1 20082024.docx [file pbio.3002895.s016.docx]

**Editor and Reviewer Comments:     

Editors Comments:**

Thank you for your patience while we considered your revised manuscript entitled "Mitochondrial calcium uptake orchestrates vertebrate pigmentation via transcriptional regulation of keratin filaments" for consideration as a Research Article at PLOS Biology. Your revised study has now been evaluated by the PLOS Biology editors, the Academic Editor and the four original reviewers.

The reviews are attached below. In light of the reviews, we are pleased to offer you the opportunity to address the remaining points from Reviewers 2 and 4 in a revision that we anticipate should not take you very long. We will then assess your revised manuscript and your response to the reviewers' comments with our Academic Editor aiming to avoid further rounds of peer-review, although might need to consult with the reviewers, depending on the nature of the revisions.

Response: We thank the Reviewers, the Academic Editor and you for the positive feedback on our revised manuscript. Our sincere gratitude towards Reviewer 1 and Reviewer 3 for accepting the revised manuscript. In this revision, we have performed all the additional experiments suggested by the Reviewer 4. Further, as recommended by Reviewer 2, we have deliberated on NFAT2 data in more detail. We genuinely hope that the Academic Editor and you will find the revised manuscript fit for final acceptance.

As mentioned, we would like you to perform the experiments suggested by Reviewer 4 as following:

1. Seahorse analysis to assess mitochondrial respiration and glycolytic activity.

Response: We would like to thank Reviewer 4 and the Editor for suggesting this experiment. In the revised manuscript, we have included data on Seahorse analysis to assess mitochondrial function. Our data suggests that MCU and MCUb do not significantly regulate mitochondrial respiration and glycolytic activity (**Supplementary Fig. 5A-I**) in B16 cells. These observations are in line with earlier data on role of MCU and MCUb in mitochondrial respiration (Baughman *et al.* Nature 2011 and Feno *et al.* Science Signaling 2021). Further, we observed that MCUb positively regulates basal respiration in these cells.

Since we do not have Seahorse instrument in our organization, we collaborated with

Drs. Anna Raffaello and Rosario Rizzuto from University of Padova for the Seahorse experiments. Therefore, Dr. Raffaello is included as an author on the revised manuscript. Please find below the Seahorse data for your quick reference:

2. ATP production assays using commercially available luminescence-based ATP assay kits.

Response: As suggested, we measured ATP production using commercially available luminescence-based ATP assay kit. Our data reveal that MCU and MCUb do not play a significant role in modulating ATP production in B16 cells (**Supplementary Fig. 5K**). These observations complement to the Seahorse analysis wherein also data on ATP production shows no significant change. Please refer to the ATP production data below:

3. Mitochondrial membrane potential assessments using TMRE or JC-1 dyes (flow cytometry or microscopy).

Response: As recommended, we assessed mitochondrial membrane potential by performing TMRE based flowcytometry experiments. This analysis demonstrate that both MCU and MCUb do not regulate mitochondrial membrane potential of B16 cells (**Supplementary Fig. 5J**). It is important to note that the seminal studies on MCU and MCUb have also reported that MCU and MCUb do not regulate mitochondrial membrane potential (De Stefani *et al.* Nature 2011, Baughman *et al.* Nature 2011 and Raffaello *et al.* EMBO 2013). Please find below the mitochondrial membrane potential data:

**Reviewers' comments**

**Reviewer 1:**

The authors significantly improved the manuscript during revisions. All of my initial critiques are being addressed. The new genetic data is very compelling and shows an important role for mitochondrial calcium signaling in regulation of cellular calcium dynamics and ensuing changes in gene expression and pigmentation.

Response: We sincerely thank the Reviewer 1 for appreciating our revised manuscript and finding it fit for publication without any further experimentation.

**Reviewer 2:**

Authors have addressed many of my concerns and the manuscript is significantly improved. However, the finding that 10 micromolar Cyclosporine is required to inhibit aMSH-induced pigmentation is clear evidence that Caclineurin-NFAT axis is not involved in this process. This conclusion is grossly overstated and the evidence is very weak. Note that Cyclosporine has an IC50 of 5 nM for Calcineurin. In T cells, 10-20 nM cyclosporine completely prevents NFAT translocation. Authors show clearly that even 100 nM cyclosporine has no effect on melanogenesis. They get an effect with 10 uM (1000-fold higher than IC50) where non-specific effects of cyclosporine, including on mitochondrial mechanisms are in play. Furthermore, the technical quality of NFAT translocation data does not inspire confidence. In control cells, Histamine resulted in practically no translocation despite Ca2+ elevations. These data are derived with transient transfection of NFAT-GFP and the images seem
cherry-picked. In light of these concerns, my view is that the conclusion of NFAT involvement is not definitive and needs to be rolled back. Authors may suggest that possibility along with the caveats noted above. This change is especially important in the abstract. Beyond this significant reservation, I do not have a problem with this manuscript moving towards publication.

Response: We thank the Reviewer 2 for acknowledging that the revised manuscript is significantly improved and we have addressed his/her concerns.

First of all, we completely agree with the Reviewer that NFAT2-kertin5 may not be the only signaling pathway that connects changes in the mitochondrial Ca^2+^ uptake to pigmentation levels. Therefore, we have clearly discussed this point in the “Discussion” section. Please find below the discussion for the quick reference:

“In this study, we have focused on understanding the role of keratin intermediate filaments in regulating pigmentation downstream of mitochondrial Ca^2+^ uptake. However, in our transcriptomics analysis we observe that several other signaling modules are differentially regulated upon MCU and MCUb silencing (Fig 4D and S7B Fig). Therefore, NFAT2-Keratin5 axis can be one of the multiple signaling cascades that work downstream of mitochondrial Ca^2+^ signaling to regulate pigmentation. Going forward, it would be worth to meticulously investigate the contribution of these differentially regulated pathways in vertebrate pigmentation.”

However, in support of NFAT2’s role in bridging mitochondrial Ca^2+^ signaling to pigmentation, apart from drug (cyclosporine) data, we have provided several other lines of evidences. They are listed/discussed below:

1. Unbiased and extensive bioinformatic analysis.

2. Significant difference in NFAT2 driven luciferase activity of WT and Mutant keratin5 promoter.

3. Luciferase assays with MyoD1 promoter (to rule out the possibility of false positive results with NFAT2 overexpression).

4. Analysis of mRNA levels of keratin5 upon NFAT2 overexpression.

5. Enhanced nuclear translocation of NFAT2 upon MCU silencing. Here, we would like to request the Reviewer to kindly look at the quantitative data from close to 100 cells/condition **(Fig 6C and 6D)**, wherein one can clearly appreciate the spread of data on NFAT2 nuclear translocation. To address the Reviewer’s concern on the nuclear translocation in the control images, *we apologize for not using enough number of arrows to pin-point the translocation in control cells. Hence, in the revised* *manuscript we have used multiple arrows to highlight the translocation in the original control and MCU silenced images.*

6. Already published literature by independent and highly respected groups. It is important to highlight that similar results have been reported in other cellular models wherein inhibition of mitochondrial Ca^2+^ uptake results in elevation of cytosolic Ca^2+^ levels and activation of Ca^2+^ regulated transcription factors such as NFAT1/4 (Yoast *et al.* JBC 2021) and NF-𝜅B (Seegren *et al.* Nature Aging 2023). In brief, just like other cell models, perturbation in mitochondrial Ca^2+^ uptake in melanocytes results in increased cytosolic Ca^2+^ levels and nuclear translocation of NFAT2.

7. Further, we thank the Reviewer to specify that “*In T cells*, 10-20 nM cyclosporine completely prevents NFAT translocation”. As discussed in the earlier revision, published literature already suggests that the effect of cyclosporine on NFAT inhibition is a cell type and context dependent phenomena. Indeed, it is already reported that higher concentration of cyclosporine is required for its function in human melanocytes (Lee et al. Pigment Cell Research 2003).

Therefore, we would like to reiterate that in light of above discussed several lines of evidences, we strongly believe that NFAT2 plays a critical role in connecting changes in mitochondrial Ca^2+^ signaling to pigmentation by regulating keratin5 transcription.

We sincerely hope that the Reviewer would be satisfied with our scientific and logical response to his/her query and would accept our revised manuscript for the publication.

**Reviewer 3:**

The authors have put forth a responsive revision and have addressed all my comments. No further comments.

Response: We genuinely appreciate the positive feedback of the Reviewer 3. We thank him/her for accepting the manuscript for publication without any further comments.

**Reviewer 4:**

The authors have revised their manuscript and successfully addressed most of the reviewers' comments. The overall quality of the paper has significantly improved.

One issue that still remains is a more accurate characterization of essential mitochondrial functions. While I am not insisting, I would like to encourage the authors to perform such experiments. After all, MCU is a mitochondrial protein complex, and characterizing its role in mitochondrial function will be beneficial for researchers working in the field.

Response: We sincerely thank the Reviewer 4 for appreciating that the overall quality of the revised manuscript is significantly improved. Further, as encouraged by the Reviewer, we have performed all the mitochondrial functional assays suggested by the Editors. In summary, our data suggests that MCU and MCUb (except basal mitochondrial respiration) do not play a significant role in these mitochondrial functions in B16 cells (**Supplementary Fig. 5**). This additional data is largely in line with earlier published data that reported a non-significant role of MCU and MCUb in regulating mitochondrial membrane potential (De Stefani *et al.* Nature 2011, Baughman *et al.* Nature 2011 and Raffaello *et al.* EMBO 2013) and mitochondrial respiration (Baughman *et al.* Nature 2011).

Furthermore, I would suggest explaining in more detail that some of the observed effects might be due to the mitochondrial modulation of SOCE and thereby NFAT activity.

Response: We thank the Reviewer for this suggestion and as recommended, we have deliberated on this point in the “Discussion” section of the revised manuscript. Please find below the discussion for the quick reference:

“Intriguingly, changes in the mitochondrial function including mitochondrial Ca^2+^ dynamics can modulate Store-Operated Calcium Entry (SOCE) mediated by plasma membrane localized Orai channels (Sekler and colleagues EMBO J 2017; Yoast *et al.* JBC 2021 and Walters *et al.* JBC, 2022). Importantly, SOCE in turn can activate and induce NFAT nuclear translocation (Feske at al. Nature 2007 and Yoast *et al.* JBC 2021). Therefore, it could be possible that downstream of changes in the mitochondrial Ca^2+^ uptake, SOCE contributes to NFAT2 activation and nuclear translocation in melanocytes. However, further studies will be required to precisely delineate the role of SOCE in connecting changes in the mitochondrial Ca^2+^ uptake with NFAT2 activation and vertebrate pigmentation.”
