# Supplementary material for: Mitochondrial calcium uptake orchestrates vertebrate pigmentation via transcriptional regulation of keratin filaments
Source: PLoS Biol. 2024 Nov 11;22(11):e3002895. doi: 10.1371/journal.pbio.3002895 (PMC11581414; doi:10.1371/journal.pbio.3002895)
Supplement: Attachment — Submitted filename: Response to Reviewers Comments Draft 1 14092024.docx [file pbio.3002895.s017.docx]

**Editor and Reviewer Comments:     

Editors Comments:**

Thank you for your patience while we considered your revised manuscript entitled "Mitochondrial calcium uptake orchestrates vertebrate pigmentation via transcriptional regulation of keratin filaments" for publication as a Research Article at PLOS Biology. This revised version of your manuscript has been evaluated by the PLOS Biology editors and the Academic Editor.

Based on our Academic Editor's assessment of your revision, we are likely to accept this manuscript for publication, provided you satisfactorily address the data and other policy-related requests stated below.

Response: Our sincere gratitude towards the Academic Editor and PLoS Biology Editors for appreciating the revised manuscript. In this minor revision, we have provided all the additional raw data suggested by the Academic Editor and the PLoS Biology team. Further, as recommended, we have made our data deposited in the NCBI Sequence Read Archive (SRA) under the accession number PRJNA1112319 publicly available. We genuinely hope that the Academic Editor and PLoS Biology team will find the revised manuscript fit for final acceptance.

**Comment 1 (Data Policy):** Many thanks for providing the data underlying the graphs shown in the figures. I have checked them and find some of the data missing. Please provide the data underlying the graphs in the following figures:

Fig. 1C, E, K, M; Fig. 2C, D, M, N; Fig. 3G; Fig. 4B, C; Fig. 5B, C; Fig. 7A, C; Fig. S2E, G, K, M; Fig. S3A; Fig. S4C, E; Fig. S5A, E and Fig. S7A, B

Response: We would like to thank the Editors regarding minor queries related to the raw data. In the revised manuscript, we have included data underlying the graphs Fig. 1C, E, K, M; Fig. 2C, D, M, N; Fig. 3G; Fig. 4B, C; Fig. 5B, C; Fig. 7A, C; Fig. S2E, G, K, M; Fig. S3A; Fig. S4C, E; Fig. S5A, E and Fig. S7A, B in S1_Data and S2_Data files. All the raw data underlying the graphs in main figures are included in S1_Data and all the raw data underlying the graphs in supplementary information are included in S2_Data.

**Comment 2 (Data Policy):** Please also ensure that figure legends in your manuscript include information on where the underlying data can be found. For example, you could add at the end of the corresponding figure legends the following: "The data underlying the graphs shown in the figure can be found in S1 Data."

Response: As recommended, we have added a line at the end of the corresponding figure legends. For example in case of Figure legend 1, we have stated that “the data underlying for panels B, C, D, E, F, H, J, K, L, M, N and P shown in the figure can be found in S1_Data”.

**Comment 3 (Data Policy):** Please also make publicly available at this time the data deposited in the NCBI Sequence Read Archive (SRA) under the accession number PRJNA1112319.

Response: As suggested we have made publicly available our data deposited in the NCBI Sequence Read Archive (SRA) under the accession number PRJNA1112319.
